# Machine Learning Models to Predict Cognitive Impairment of Rodents Subjected to Space Radiation

**DOI:** 10.3389/fnsys.2021.713131

**Published:** 2021-09-13

**Authors:** Mona Matar, Suleyman A. Gokoglu, Matthew T. Prelich, Christopher A. Gallo, Asad K. Iqbal, Richard A. Britten, R. K. Prabhu, Jerry G. Myers

**Affiliations:** ^1^NASA Glenn Research Center, Cleveland, OH, United States; ^2^ZIN Technologies, Inc., Cleveland, OH, United States; ^3^Department of Radiation Oncology, Eastern Virginia Medical School, Norfolk, VA, United States; ^4^Universities Space Research Association, Cleveland, OH, United States

**Keywords:** space radiation, radiation research, behavioral decrement, cognitive impairment, impairment prediction, rodent studies, machine learning, artificial neural network

## Abstract

This research uses machine-learned computational analyses to predict the cognitive performance impairment of rats induced by irradiation. The experimental data in the analyses is from a rodent model exposed to ≤15 cGy of individual galactic cosmic radiation (GCR) ions: ^4^He, ^16^O, ^28^Si, ^48^Ti, or ^56^Fe, expected for a Lunar or Mars mission. This work investigates rats at a subject-based level and uses performance scores taken before irradiation to predict impairment in attentional set-shifting (ATSET) data post-irradiation. Here, the worst performing rats of the control group define the impairment thresholds based on population analyses via cumulative distribution functions, leading to the labeling of impairment for each subject. A significant finding is the exhibition of a dose-dependent increasing probability of impairment for 1 to 10 cGy of ^28^Si or ^56^Fe in the simple discrimination (SD) stage of the ATSET, and for 1 to 10 cGy of ^56^Fe in the compound discrimination (CD) stage. On a subject-based level, implementing machine learning (ML) classifiers such as the Gaussian naïve Bayes, support vector machine, and artificial neural networks identifies rats that have a higher tendency for impairment after GCR exposure. The algorithms employ the experimental prescreen performance scores as multidimensional input features to predict each rodent’s susceptibility to cognitive impairment due to space radiation exposure. The receiver operating characteristic and the precision-recall curves of the ML models show a better prediction of impairment when ^56^Fe is the ion in question in both SD and CD stages. They, however, do not depict impairment due to ^4^He in SD and ^28^Si in CD, suggesting no dose-dependent impairment response in these cases. One key finding of our study is that prescreen performance scores can be used to predict the ATSET performance impairments. This result is significant to crewed space missions as it supports the potential of predicting an astronaut’s impairment in a specific task before spaceflight through the implementation of appropriately trained ML tools. Future research can focus on constructing ML ensemble methods to integrate the findings from the methodologies implemented in this study for more robust predictions of cognitive decrements due to space radiation exposure.

## Introduction

The current record for the longest single flight a NASA astronaut spent on the International Space Station is 340 days (NASA, 2020), and travelers aboard are partially shielded from galactic cosmic radiation (GCR) and Solar Particle Events by Earth’s magnetic field (Chancellor et al., 2014). Missions to Mars will last about 3 years, the majority of which occurring beyond the magnetosphere. Astronauts are expected to receive close to 40 cGy of GCR based on the assessment of data obtained from Curiosity’s radioisotope thermoelectric generator onboard a trip to Mars in 2011 (Zeitlin et al., 2013).

Animal surrogate models are commonly used as ground-based analogs to quantify the effects of single and multiple GCR ions on astronauts’ operational performance. This type of study helps NASA in defining human permissible outcome limits (POLs)^1^ and space permissible exposure limits (SPELs)^2^ for future mission success (NASA, 2015). Several studies show wide-ranging health problems in rodents triggered by ionizing radiation exposure. In these animal model studies, cancer (Cucinotta et al., 2011; Sridharan et al., 2015; Blattnig and Chappell, 2016), cardiovascular issues (Baker et al., 2011; Boerma, 2015), and central nervous system (CNS) damage (Rabin et al., 2005; Cucinotta et al., 2014; Parihar et al., 2015; Cekanaviciute et al., 2018) represent pathologies frequently attributed to a deleterious ionizing radiation dose. Moreover, exposure to high-energy protons, or high-atomic number and energy (HZE) ions produces CNS damage leading to decremental behavioral changes in rodents (Rabin et al., 1998, 2015; Britten et al., 2012, 2014, 2020, 2018; Lonart et al., 2012; Haley et al., 2013; Cucinotta et al., 2014; Hadley et al., 2016; Parihar et al., 2016, 2018; Blackwell et al., 2018; Carr et al., 2018; Jewell et al., 2018; Mange et al., 2018; Shukitt-hale et al., 2018; Kiffer et al., 2019) and mission success, as spaceflights require constant alertness and readiness to address critical situations. Many studies utilize the novel object recognition, Barnes maze, Morris water maze, active avoidance, open field, or operant responding as the appropriate behavioral assays to evaluate rodents’ cognitive impairment (Rabin et al., 1998, 2011, 2015; Britten et al., 2012; Lonart et al., 2012; Haley et al., 2013; Hadley et al., 2016; Parihar et al., 2016, 2018; Carr et al., 2018; Jewell et al., 2018; Shukitt-hale et al., 2018; Kiffer et al., 2019). Another test that assesses executive cognitive functions in rats is the attentional set-shifting (ATSET) test (Birrell and Brown, 2000; Young et al., 2010), which is a seven-stage test analog to the human Wisconsin Card-Sorting Test (WCST) (Grant and Berg, 1948; Eling et al., 2008). The WCST estimates cognitive flexibility such as attention, perseverance, working memory, abstract thinking, and set-shifting.

Previous work reveals cognitive deficits in rats during different stages of the ATSET depending on the ion type and dose of radiation exposure. Performance in the simple discrimination (SD) stage is impaired after 5 cGy of ^28^Si (Britten et al., 2018), compound discrimination (CD) and compound discrimination reversal (CDR) after 20 cGy of ^48^Ti (Hadley et al., 2016), and SD and CD after ≤15 cGy of ^56^Fe or ≤10 cGy of ^4^He (Jewell et al., 2018; Burket et al., 2021). These decrements and most behavioral deficits in the studies listed above are reported as a change in the irradiated cohort’s test score from the corresponding non-irradiated group’s score (Rabin et al., 2005, 2011, 2012, 2019, 2015; Cucinotta et al., 2014; Acharya et al., 2015; Parihar et al., 2015; Cekanaviciute et al., 2018; Shukitt-hale et al., 2018; Britten et al., 2020). We define this non-irradiated group as sham. This difference is represented using bar plots with means and standard error of means, accompanied by a statistical *t*-test such as the Mann–Whitney to specify the significance of the difference between groups. While these statistical tests and plots are valuable, given the low sample size in such studies and the extent to which an affected individual score impacts the skewness of the population data, seen as the mean of the sample scores deviating significantly from their median, these averages alone likely represent an inadequate characterization of the change in the overall population performance. Furthermore, inter- and intra-individual variabilities in responses to brain stimulation paradigms (Fiske and Rice, 1955; Loevinger, 1957; López-Alonso et al., 2014; Wyrobek and Britten, 2016; Sherman and Sladky, 2018) lead to inconsistent means across sample populations and disqualify their inferences on the general population.

The application of machine learning (ML) techniques in clinical psychology and psychiatry provides a means to predict impairment in people, such as in Alzheimer disease, given the multidimensional records provided for each patient (Yu et al., 2015; Long J. et al., 2016; Long Z. et al., 2016; Balagopalan et al., 2020). Rather than subject based ML techniques, space radiation, cognitive impairment studies typically show results from traditional statistical analyses and infer conclusions on subjects belonging to parallel groups being irradiated (Rabin et al., 2005, 2011, 2012, 2019, 2015; Cucinotta et al., 2014; Acharya et al., 2015; Parihar et al., 2015; Cekanaviciute et al., 2018; Shukitt-hale et al., 2018), or using some biophysics-based models at the neuron level to describe changes in morphology that can affect cognition (Alp and Cucinotta, 2018; Cacao et al., 2018). However, given the abundance of data collected during such studies, it is possible that multidimensional feature data about each subject before radiation exposure is sufficient to build robust data-driven approaches for predicting post-irradiation impairment at the subject level.

The current study uses data obtained from the assessment of the performance of rat models in the ATSET test before and after irradiation with a single GCR ion beam (^4^He, ^16^O, ^28^Si, ^48^Ti, and ^56^Fe) (Lonart et al., 2012; Britten et al., 2018). Statistical methods are applied to the experimental behavioral data to determine thresholds for impairment based on the performance of the sham rats as a group. Then, ML techniques with potential to discriminate the uniqueness of each subject are utilized to predict their impairment after irradiation. The ML model learns the cognitive aspects of each rat based on the corresponding prescreen ATSET scores, which are the scores calculated during a pre-selection process before irradiation. The ML algorithms implemented in this study are support vector machines (SVM) that take a geometric approach to draw spatial boundaries between different classes, Gaussian naïve Bayes (GNB) that use a probabilistic approach based on Bayes Rule, and artificial neural networks (ANNs) that allow iterative training through components of the network to better fit the data on hand. Since each of these ML classifiers has a different underlying mathematical underpinning, each might capture existing unique patterns in the data. Based on the ion/dose given during irradiation, the model predicts the rat impairment 3 months post-irradiation.

The paper is structured as follows. First, details about the adopted statistical tools and ML algorithms are given in the “Materials and Methods” section. This section includes an in-depth description of the various parameters explored and the cross-validation techniques used to address the class imbalance, mitigate the small sample size, and generalize the analysis to independent validation data. Next, we apply our methods to the data, evaluate the prediction performance of our model, and present the findings of our modeling efforts in the “Results” section. Lastly, we discuss the results and describe the benefits of the current *in silico* analyses and potential impact for crewed Lunar and Mars missions in the “Discussion” section. Limitations of the current modeling efforts are addressed along with viable paths for future work.

## Materials and Methods

**FIGURE 1:**
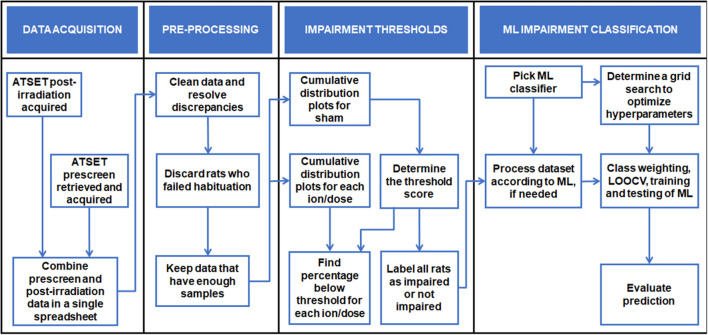
Visualization of the ML procedures as a guide to follow and locate specific algorithms within the text.

The work in this paper follows the computational analysis process illustrated in Figure 1, which serves as a visual guide to locate specific algorithms within the text. This section describes the behavioral test generating the data used, the datasets with their pre-processing, and the different computational tools employed to achieve the ultimate goal of determining which rats are impaired after irradiation. In the materials and methods, we follow NASA STD-7009, a technical standard for modeling and simulation practices related to NASA’s mission. We follow suggested rules for the credible practice of modeling and simulation in healthcare to ensure data pedigree, results robustness and limitations, and model reproducibility (NASA, 2019; Erdemir et al., 2020).

### Behavioral Test

Data used in this paper is collected from a behavioral experiment, known as ATSET, performed by Dr. Richard Britten’s research group at Eastern Virginia Medical School (EVMS) (Norfolk, VA, United States). Subjects (*N* = 432) are male Wistar rats, about 10-month old at the time of irradiation, maintained on a reversed 12:12 light-dark schedule and kept on a strict food and exercise regimen for the entire duration of the study to match the physical fitness imposed on astronauts (Lonart et al., 2012; Hadley et al., 2016; Wyrobek and Britten, 2016; Britten et al., 2018; Jewell et al., 2018). Rats are group-housed and have *ad libitum* access to autoclaved chow and municipal water by bottle till around 7 days prior to the start of ATSET paradigm, when they are single-housed and switched to a food diet of 6 g of Cheerios^TM^ (General Mills, Minneapolis, MN, United States) per day, varying the amount on a daily basis to maintain each subject at 85% of its weight on the first day of the testing. Their exercise regimen consists of gradually increasing training on a motor treadmill (Véras-Silva et al., 1997) to reach a maintenance regimen of 30 min at 25 m/min, twice a week, for the entire duration of the study (except when at the irradiation lab and during the week of ATSET testing, both prescreen and post-irradiation phases).

The ATSET is a constrained cognitive flexibility test consisting of seven stages where the rats forage for a food reward buried in a bowl associated with a digging media and/or scent depending on the stage (Shepp and Eimas, 1964). These stages, in successive order, are SD, CD, CDR, intra-dimensional shifting (IDS), intra-dimensional shifting reversal (IDR), extra-dimensional shifting (EDS), and extra-dimensional shifting reversal (EDR) (Lonart et al., 2012; Hadley et al., 2016). Depending on the skills required at each stage, different cognitive skills are required; therefore, different brain regions might be activated. In each stage, the food reward is buried four cm in the digging media within one of two bowls, and each bowl is associated with a scent and digging media. Depending on the stage, one cue is relevant, and the other is not, and the rat learns to associate the reward with the relevant cue. A list of the of rewarded and non-rewarded associative cues for both prescreen and post-irradiation screening is in Tables 1, 2 of the published work (Jewell et al., 2018). At each stage, rats are given up to 30 trials to get the food reward, for at most 3 min each trial, and are required to succeed in six consecutive attempts in order to reach the criterion. If a rat does not reach the criterion or the score is incomplete, the rat is rested overnight and re-tested the following day. Each stage is granted at most 2 days.

**TABLE 1:** Sample size at the first stage of the ATSET post-irradiation for each ion/dose combination provided by Dr. Richard Britten’s research group by March 2020 and is inclusive of the experimental results previously published [Parihar et al., 2016 (Ti); Jewell et al., 2018 (Fe); Britten et al., 2020 (Si); Burket et al., 2021 (He)].

Ion\Dose(cGy)	0	1	1.5	3	5	10	15
Sham	62						
^4^He		19			20	15	
^16^O			6		18	11	
^28^Si		15		14	63	45	15
^48^Ti				8	16	12	10
^56^Fe		9		15	26	17	16

*“Sham” is used to indicate non-irradiated rats.*

**TABLE 2:** Performance of classifiers when predicting impairment of sham and 1, 5, or 10 cGy ^56^Fe irradiated rats in the post-irradiation ATRC scores of the SD and CD stages.

Impairment threshold	ML algorithm	% Accuracy	AUC-ROC	AUC-PR	MCC	F_1_ score
SD ATRC ≥ 16	GNB	66	0.68	0.33	0.21	0.35
	SVM	81	0.65	0.37	0.30	0.41
	ANN	71	0.69	0.37	0.25	0.38
CD ATRC ≥ 36	GNB	72	0.64	0.28	0.30	0.42
	SVM	74	0.58	0.30	0.07	0.22
	ANN	75	0.66	0.32	0.30	0.43

*The input features are the dose (0, 1, 5, or 10 cGy) and the prescreen ATRC scores.*

In both the prescreen and post-irradiation procedures, and before the SD stage, an additional habituation (HAB) stage is carried out where the rats get accustomed to the test maze and the handlers and are trained to forage for a food reward. In that stage, rats are required to dig for their reward of Cheerios placed in the digging medium with no scent and at different depths. Those that passed are rested overnight before being tested for their ability to conduct SD. Those that fail to retrieve the buried reward are excluded from further analysis. All testing is conducted during the dark cycle, keeping the experiment time fixed for each individual rat.

The prescreen ATSET is conducted before rats are sent to BNL for irradiation, and only the first four stages are administered. The associative cues used in these stages are all scents. Any rat failing two or more stages in that test is dropped from further consideration. Furthermore, as described below, only those that pass the prescreening phase qualify for the study, similar to the high level of cognitive ability anticipated to become an astronaut. All irradiation is administered to the whole rat body at NASA Space Radiation Laboratory, Brookhaven National Laboratory (NSRL, BNL), and rats are placed in a well-ventilated custom-made irradiation jig constructed of black polyacrylic plastic. Sham rats are placed in an identical irradiation jig that remained in the preparation room. Table 1 lists the single-beam ion type and dose, along with each sample size.

**FIGURE 2:**
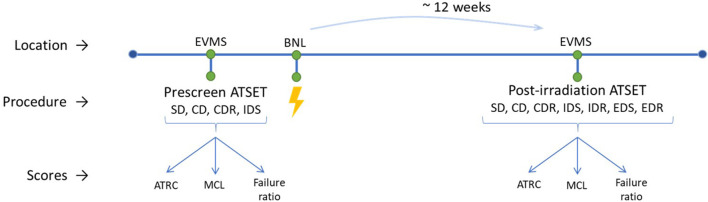
Lab experimental timeline for each run. ATSET stages are listed in the performed order. Initial findings from generated data was previously published [Parihar et al., 2016 (Ti); Jewell et al., 2018 (Fe); Britten et al., 2020 (Si); Burket et al., 2021 (He)].

The post-irradiation ATSET, including all seven stages, is conducted 12 ± 2 weeks after irradiation. The timeline is shown in Figure 2. The cues in this step are flipped from those in the prescreening since the test is for cognitive flexibility rather than memory, and cues can be found in published work (Jewell et al., 2018).

### Data

The dataset examined in this work is provided by the experimental lab in a tabular form. Initial findings on post-irradiation data was previously published (Parihar et al., 2016; Jewell et al., 2018; Britten et al., 2020; Burket et al., 2021) but prescreen scores are specifically retrieved for this study. Data is available through Dr. Britten.

#### ATSET Scores Columns

For each stage of the ATSET, the number of correct and incorrect attempts to reach the criterion are recorded in the lab, along with the time it takes for each correct attempt. The data analysis carried out in this paper utilizes three derived scores:

• The number of attempts to reach criteria (ATRC), which is the sum of correct and incorrect attempts. If a rat does not reach the criterion of a stage on the first day, the ATRC score is 30 plus the total number of attempts on the second day (Lonart et al., 2012; Britten et al., 2014, 2016, 2018; Hadley et al., 2016; Jewell et al., 2018).
• The failure ratio (FR), which is the ratio of incorrect attempts to total number of attempts, including the first and second days. The FR score is calculated to differentiate between rats having similar ATRC but a different ratio of incorrect attempts in ATRC.
• The mean correct latency (MCL) time for each rat, which is calculated as the average time it takes for all successful attempts. If a rat fails the first day, then MCL considers only the second day’s successful attempts.

Each of these three scores has a smaller value for higher performer rats. The highest scores correspond to impaired rats (see section “Step 1: Impairment Thresholds”). A pass/fail score for the HAB stage is also recorded, but since this study considers only the rats that passed habituation, this column is omitted from the datasets.

#### Ion and Dose Columns

After the prescreen at EVMS lab, the rats receive a single-ion whole-body irradiation at BNL. The number of rats exposed to each dose is given in Table 1. Sham rats have a dose of 0.

#### Data Pre-processing and Visualization

The sample sizes in Table 1 are for the SD stage in the ATSET and typically get smaller for subsequent stages due to the fact that a condition on earlier days of the test may prevent the rats from proceeding in the experiment when they fail two stages. Therefore, as part of the data pre-processing, a sample is omitted if the computational algorithm requires all stages. ^4^He, ^28^Si, and ^56^Fe data present the most likely successful opportunity for comparison as the dataset is complete for doses of 1, 5, and 10 cGy; hence, these are the ions and doses that will be considered in the model.

### Population Analyses

This section describes the population statistical analyses performed on the data. The results aggregate data points and indicate a group behavior, not necessarily representative of each rat in the group.

#### Data Visualization

A common group visualization method that researchers utilize to depict qualitative trends in groups is a bar plot presenting the means and the standard error of the means in each data group. Bar plots for post-irradiation ATRC scores in all ATSET stages are presented in this paper, with indication to which groups are significantly different from the sham group via the Mann–Whitney test.

Similarly, box plots qualitatively emphasize the variability of scores within a cohort.

#### Cumulative Distribution

A cumulative distribution function (CDF) informs the percentage of subjects that attained a certain score. It is the area under the curve (AUC) of the density function. The cumulative distribution plots have an *x*-axis holding values of an ATSET score and a *y*-axis having the percent of rats that reached the criterion with that or a lower score value. The current research utilizes these plots to derive impairment thresholds as described below.

### Two-Step Approach to Predict Subject Impairment

The training and cognitive assessment of astronauts happen on Earth away from the GCR effect. In parallel, the sham group of rats are presumed the role model in the datasets, and irradiated rats that have ATSET scores comparable to the sham group are considered non-impaired. From a computational perspective, an impaired subject is one that has performance metrics that are less than those seen in the worst performers in the sham cohort. The choice of the threshold for impairment depends on what percentage of the control group is considered operating at acceptable capacity. This research takes the impairment at 5% worst performers in the non-irradiated cohort, or stated differently, at 95% impairment. After the threshold is determined, each rat is labeled as impaired or not based on their score. The dataset is fed to an ML classifier to learn an association between the input and output features. Here, the input features are GCR ion dose and the prescreen scores, and the output feature is the impairment label associated with cognitive performance post-radiation.

#### Step 1: Impairment Thresholds

The impairment threshold is determined based on the percentage of samples in the sham group that are considered “normal” or non-impaired. A population statistical analysis finds the threshold that separates them from the impaired rats. This work utilizes the CDF described in section “Cumulative Distribution.” The score at or below which 95% of the sham group pass a stage defines the impairment threshold score.

#### Step 2: Predict Impairment via ML

Machine learning classifiers predict impairment at a subject-based level. Having data carrying a label suggests using supervised ML techniques. Data samples are split into two subsets, a training set and a testing set. The training set first provides the classifier with the given dose and some prescreen scores as input features and the impairment label as target feature. The classification is referred to as binary since the output belongs to one of two classes: the positive class (class of impaired), or the negative class (class of non-impaired). Each classifier follows a specific ML algorithm to learn an association between the input features and the target feature and adjusts the parameters of the model to accommodate the given samples; this phase is called the “training” phase. Then the classifier receives the second unseen subset of data, i.e., the testing set, with only the input features, and predicts the output feature; this concludes the “testing” phase. A comparison between the predicted values in this stage and the actual impairment labels informs the classifier reliability. For ML methods, a third validation set is ideal, but a common practice for data with a small sample size, as is the case here, is to use a cross-validation technique to establish model predictive characteristics, as described in section “Cross-Validation, Class Weighting.”

Below are various ML classifiers utilized in this study. Scikit-Learn for Python is the ML library used to generate the results in this paper (Pedregosa et al., 2011; van Rossum and The Python Development Team, 2018).

#### Evaluating ML Performance

Many metrics are available to evaluate the performance of each ML model. They are based on comparing the predicted impairment labels in the testing phase and the actual output impairment labels of rats. A review of these metrics and some useful definitions are below (Fawcett, 2004; Hossin and Sulaiman, 2015; Tharwat, 2018):

• True positive (TP): the number of impaired samples that are correctly classified as impaired by the ML-based model.
• True negative (TN): the number of non-impaired samples, which are correctly classified as non-impaired by the ML-based model.
• False positive (FP): the number of non-impaired samples that are wrongfully classified as impaired by the model.
• False negative (FN): the number of impaired samples that are wrongfully classified as non-impaired by the model.
• True positive rate (TPR) = $\frac{TP}{TP+FN}$, and varies from 0 for not classifying any positive correctly to 1 for classifying all the positives correctly.
• False positive rate (FPR) = $\frac{FP}{FP+TN}$, and varies from 0 for not misclassifying any negative as positive to 1 for misclassifying all negatives as positives.
• Accuracy = $\frac{\left(TP+TN\right)}{TP+TN+FP+FN}$, and varies from 0 for not classifying any negative or positive correctly to 1 for classifying all of them correctly. Accuracy is the most common metric to evaluate a classifier. However, when the data is highly imbalanced, the accuracy tends to lean to the majority class.
• Precision = $\frac{TP}{TP+FP}$, and varies from 0 for not classifying any positive correctly to 1 for not misclassifying any negative as positive. An increase in precision indicates a decrease in the number of false positives.
• Recall = TPR = $\frac{TP}{TP+FN}$. An increase in recall indicates a decrease in the number of false negatives, i.e., it is less likely that a positive prediction is missed. Different models offer different trade-offs between precision and recall evaluations. It is more important to increase the recall at the expense of precision when it is more important to classify a positive even when this yields more false positives.
• The precision-recall (PR) curve holds the recall on the *x*-axis and precision on the *y*-axis. A PR curve is produced by plotting multiple PR pairs when varying a model’s classification cutoff value; this is the number between 0 and 1 that determines if an output feature leads to a positive or negative classification label.
• The AUC of PR curve is a measure that can be used to compare the performance of multiple classifiers based on their precision and recall curves. In general, a higher AUC-PR indicates a better classifier. A random guess yields an AUC-PR equal to the ratio of the sample size of the minority class, which, in this work, is the number of impaired rats divided by the total number of rats.
• The receiver operating characteristic (ROC) curve holds FPR on the *x*-axis and TPR on the *y*-axis. Just like the PR curve, the ROC curve is built by varying the cutoff classification value that separates positives from negatives. A random guess is represented by a diagonal straight line going from the bottom left to the top right of the plot, and a “better-than-chance” classifier gives FPR-TPR pairs that sit above this line.
• The AUC of ROC curve can be used to compare the performance of multiple classifiers based on their TPR and FPR. The model is generally better when the AUC-ROC is higher. A random guess yields an AUC-ROC of 0.5.
• F_1_ = $\frac{2\cdot Precision\cdot Recall}{Precision+Recall}$, the harmonic mean between precision and recall is the square of the geometric mean divided by the arithmetic mean. This metric serves as a comparative tool between classifiers and varies from 0 if no true positives are detected to 1 for perfect classification.
• Matthews correlation coefficient (MCC) = $\frac{TP\cdot TN-FP\cdot FN}{\sqrt{\left(TP+FP\right)\left(TP+FN\right)\left(TN+FP\right)\left(TN+FN\right)}}$, varies between −1 for total disagreement between predictions and actual labels and 1 for perfect prediction. A random guess has an MCC of 0.

### ML Algorithms

#### Gaussian Naïve Bayes

Gaussian Naïve Bayes is a probabilistic classifier that is based on Bayes conditional independence rule. The probability that a testing sample belongs to a certain output class depends on probabilities deduced from the samples in the training set.

A simple way to understand the algorithm is as follows. Given a testing sample with some input features like dose and prescreen scores, for each possible output class (here classes are negative: non-impaired and positive: impaired), the probability of belonging to the class is:


$$Pr\left(class|input\right)=\frac{\text{Pr}\left(class\right)}{\text{Pr}\left(input\right)}\prod_{i=1}^{\text{n}}\mathrm{Pr}\left(inputfori^{\text{th}}sample|class\right).$$

The sample in the testing data is assigned the class with the largest predicted probability given the corresponding input features (Micheal, 1997; Rish, 2001). This research uses uniform priors for the GNB model, starting the untrained classifier by assuming a 50% chance for either one of the two output classes.

#### Support Vector Machines

Support vector machine is an ML classifier that uses a geometric approach to separate samples based on their label, i.e., their output feature class. It builds a hyperplane in the input features space to maximize the margin of separation among classes of the output feature. The hyperplane is linear if the data is separable. The hyperplane is non-linear when using a kernel that transforms the data into a higher dimensional space where the classes are linearly separable, then transforms it back to the original feature space. This study shows results using the Gaussian radial basis function as a kernel (Boser et al., 1992). The testing phase consists of placing the samples of the testing set in the input feature space and deciding on their class based on their geometric location on either side of the hyperplane (Boser et al., 1992; Noble, 2006).

#### Artificial Neural Networks

**FIGURE 3:**
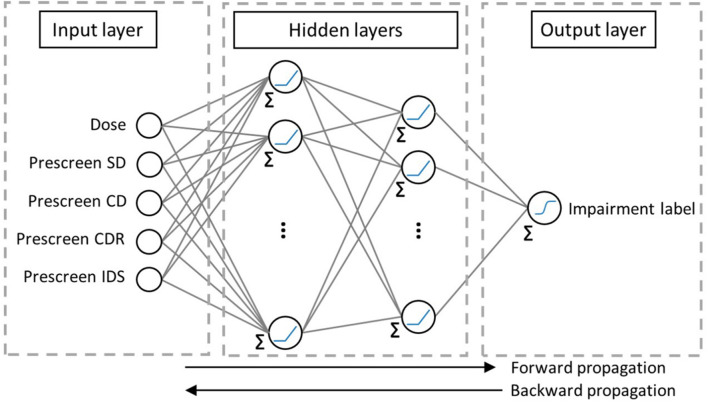
The ANN structure used in this study. The first hidden layer contains 16 nodes and the second one has 8. Each link has a weight. “Σ” before a node indicates that its input is the sum of the weighted outputs from previous nodes. “
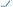
” refers to a ReLU activation function and “
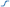
” refers to the Sigmoid.

Artificial neural networks are inspired by the biological learning and memory retention in the system of interconnected neurons. ANNs are built from layers of neurons called *nodes* and links between neurons. Each of the nodes stores an *activation function* specified by the user. Each of the links between nodes (inspired by neuron connectivity) from a layer to the adjacent one is associated with a weight that gets adjusted at each back-propagation step, as in Figure 3. The first layer of nodes called the *input layer*, stores the input features, the last layer, called the *output layer*, gives the predicted class, and the in-between layers are called the *hidden layers*. Information from the input features of the training samples travel from the input to the output layers, where each node, except those in the input layer, receives numbers from the connected nodes in the previous layer, multiplies them by the links’ weights, sums them up, and applies an activation function to pass one number to the nodes in the next layer. At the output layer, the algorithm compares the predicted value to the sample’s output feature and assesses the error over the training set. It then propagates backward to adjust its weights and repeats the process until it reaches a stopping criterion designed by the user. The testing set’s input features feed into the ANN, and their output is predicted and compared to their actual label in order to quantify the algorithm’s performance by some metric such as the accuracy, AUC-ROC, AUC-PR, MCC, and F_1_ score. More information about the variations in types of ANNs can be found in the literature (Hecht-nielsen, 1992; Micheal, 1997; Goodfellow et al., 2016).

In the current effort, due to the small sample size (see Table 1), the ANN is feed-forward with one hidden layer of sixteen nodes and another of eight nodes each harvesting a *Rectified Linear Unit* (*ReLU*) activation function. The output layer applies a *Sigmoid* activation function to predict values between 0 and 1, translatable into the sample’s predictable probability of impairment.

#### Cross-Validation, Class Weighting

Through all the ML classifiers implemented in this work, it is necessary to take measures to accommodate the low sample size and the class imbalance in the data, along with finding the optimal hyperparameters for each model.

**FIGURE 4:**
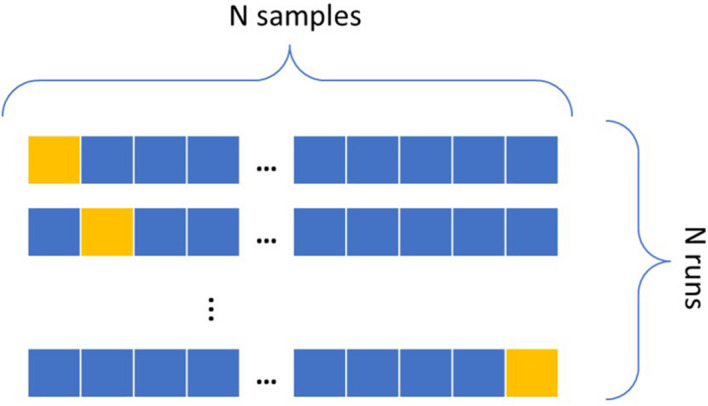
Leave-One-Out Cross-Validation. The blue samples consist of the training set, and the yellow one is the testing sample. This runs N times leaving a different testing sample out for each run.

To make the most use of the small sample size in the dataset, Leave-One-Out Cross-Validation seen in Figure 4 is implemented, where all samples except one serve as the training set and the remaining one sample as the testing set. For a dataset of N samples, the cross-validation refers to repeating this process N-1 times, each time leaving a different sample out for testing (Efron, 1982). An ML algorithm learns an association between the input and output features by inspecting the samples of the training set and optimizing an error function to improve a classification metric, typically the accuracy. For an imbalanced dataset, i.e., a dataset that contains the majority of datapoints belonging to one class (the negative class in this case), a high accuracy can be achieved by simply labeling all samples as belonging to the class with the largest sample size. The dataset in this research includes less impaired rats than non-impaired. Take, for example, the sham rats, labeling the worst 5% performers as impaired; a classifier can resort to allocating all of them to the negative class for a 95% accuracy, thereby missing all impaired rats. When translating such result to astronauts, the impairment of the minority of impaired astronauts, or the positive class, is totally dismissed. Due to the severity of the risk that an impaired astronaut can pose to a mission’s success, identifying individuals in the positive class is of high interest. This issue is also common in applications outside of space behavior, such as in fraud detection or illness detection (Di Martino et al., 2012; Wang et al., 2016; Johnson and Khoshgoftaar, 2019). A popular solution is to assign a different weight to each class, giving higher importance to the minority class to reduce bias toward the majority class.

## Results

**FIGURE 5:**
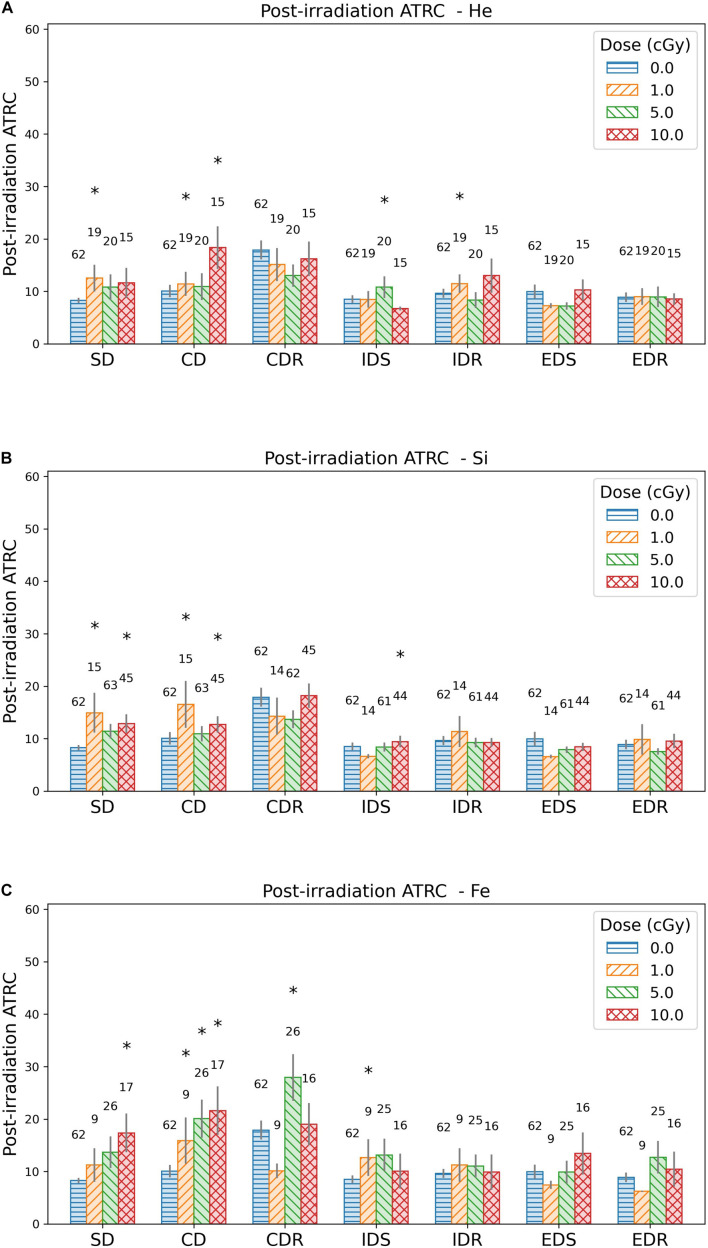
Bar plots showing the mean and standard error of the means of ATRC scores for different ATSET stages post-irradiation with **(A)** ^4^He, **(B)** ^28^Si, or **(C)** ^56^Fe ions. The numbers above the bars are the sample size. Asterisks refer to groups that are significantly different from the sham group using the Mann–Whitney test.

**FIGURE 6:**
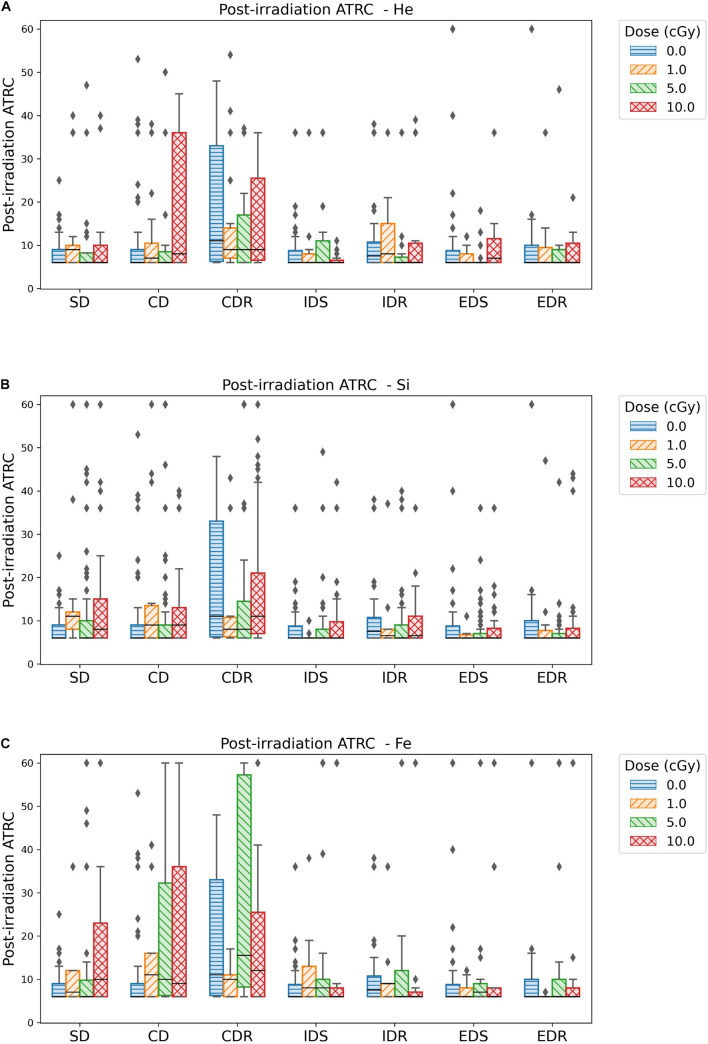
Box plots showing the median, quartiles, and outliers of ATRC scores for different ATSET stages post-irradiation with **(A)** ^4^He, **(B)** ^28^Si, or **(C)** ^56^Fe ions. Fifty percent of the data falls within, 25% above, and 25% below the box. The median, which is the middle score, is the horizontal line inside the box. The diamonds or individual data points far from the box represent the outliers.

Experimental data of the rodent ATSET performance are plotted in Figures 5, 6. These graphs compare sham and each group irradiated with one of ^4^He, ^28^Si, and ^56^Fe ions at a dose of 1, 5, or 10 cGy. Next, cumulative distribution plots depict the threshold corresponding to 95% of the sham population in each of the seven stages of the post-irradiation test for the ATRC and MCL scores. The current study focuses on analyzing ATRC scores of the SD and CD stages. Rats scoring at or above the threshold are labeled impaired while the others are non-impaired. The third part of this section employs the four stages of prescreen ATSET test scores and the dose applied and implements GNB, SVM, and ANN classifiers to predict post-irradiation impairment in the SD and CD stages.

### Post-irradiation ATRC Data Visualization

Attempts to reach criteria scores are visualized in Figure 5 as bar plots for the sham group and those irradiated with ^4^He, ^28^Si, or ^56^Fe ions, showing means and associated standard error of the means. The statistically significant difference is ascertained using the Mann–Whitney *U* test. Stages CDR, IDS, and IDR for ^4^He, ^28^Si, and ^56^Fe irradiated rats show at most one dose group to be significantly different from the corresponding sham, and stages SD and CD show a significant difference in at least one dose group. We also note in Figures 5A–C that an applied dose of 10 cGy of any of the three ions shows a significant difference from shams in the ATRC scores of the CD stage. In addition, a dose-effect is prominent after exposure to ^56^Fe ion in the SD and CD stages.

Bar plots for MCL and FR scores are in the Supplementary Tables. They show a less significant difference in MCL scores than ATRC but a difference in FR scores comparable to the difference in ATRC when performing the Mann–Whitney *U* test.

Figure 6 box plots show how data are spread out in the different quartiles. These plots show the large variance in the CDR stage for the sham rats and a higher median than its counterpart for the groups irradiated with any dose of ^4^He (Figure 6A) or ^28^Si (Figure 6B) ions. This suggests that ML is not suitable for the CDR stage due to the high variability in performance among rats.

Based on the above observations, we choose to build our ML predictive models using post-irradiation ATRC scores in the SD and CD stages to measure behavior after exposure.

### Cumulative Distributions and Impairment Thresholds

**FIGURE 7:**
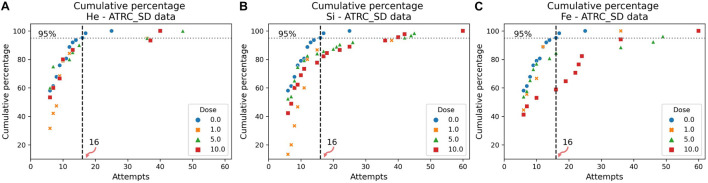
Cumulative distribution plots of the ATRC scores in the SD post-irradiation stage for rats irradiated with **(A)** ^4^He, **(B)** ^28^Si, or **(C)** ^56^Fe ions. The vertical dashed line shows the impairment threshold at 16 when ruling 95% of the sham population (in blue) as non-impaired.

**FIGURE 8:**
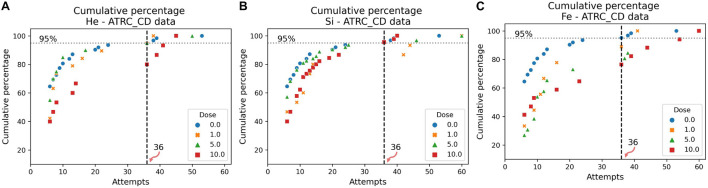
Cumulative distribution plots of the ATRC scores in the CD post-irradiation stage for rats irradiated with **(A)** ^4^He, **(B)** ^28^Si, or **(C)** ^56^Fe ions. The vertical dashed line shows the impairment threshold at 36 when ruling 95% of the sham population (in blue) as non-impaired.

The cumulative distribution plots of the sham data, in Figures 7, 8, show the results of the evaluation procedure for an impairment threshold for an SD or CD stage. In these plots, the *y*-axis refers to the percent of rats that reached the criterion in at-most the number of attempts on the *x*-axis. The horizontal “dashed” line identifies the cumulative 95% level and the vertical line, associated with the 0 cGy dose (blue circles, sham data), identifies the impairment threshold. The impairment threshold is 16 for the SD post-irradiation stage as seen in Figure 7 and 36 for the CD post-irradiation stage in Figure 8. These thresholds determine the impairment label of the samples used in the supervised ML techniques of the following section.

**FIGURE 9:**
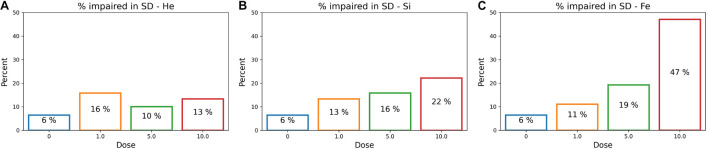
The percentage of impaired rats in the post-irradiation SD stage for an ATRC impairment threshold of 16. Colors refer to different doses. Plots are for **(A)** ^4^He, **(B)** ^28^Si, or **(C)** ^56^Fe irradiated rats.

**FIGURE 10:**
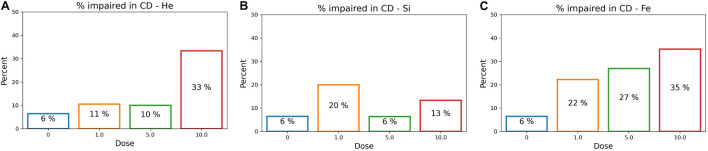
The percentage of impaired rats in the post-irradiation CD stage for an ATRC impairment threshold of 36. Colors refer to different doses. Plots are for **(A)** ^4^He, **(B)** ^28^Si, or **(C)** ^56^Fe irradiated rats.

After determining an impairment threshold, the percentage of rats that are impaired is calculated for different ions at a dose of 1, 5, or 10 cGy, and results are plotted in Figure 9 for the SD stage and Figure 10 for the CD stage. These plots can be interpreted as the probability of a rat exhibiting impairment or susceptibility to impairment. More details about these results and their implication are in the “Discussion” section.

Following the same framework implemented for ATRC scores, the MCL score’s threshold of impairment is 53 in the SD stage and 72 in the CD stage. The cumulative distribution MCL plots (not shown here) do not show a higher percentage of impaired rats for most irradiated groups.

As for the FR scores described in section “ATSET Scores Columns,” defining an impairment threshold for sham rats does not serve as a good baseline for the behavior of shams, as two very differently performing rats can have the same FR. For example, a rat that reaches criterion in nine attempts, where it gets three wrong and six correct, gets the same FR score of 1/3 as a rat that reaches the criterion in 24 attempts by successively getting five correct and one wrong three times in a row, until finally learning and getting six correct attempts for criterion. We therefore proceed the analyses without using these scores.

All ATRC and MCL impairment scores are reported in Supplementary Table 1, for the seven stages of post-irradiation ATSET.

### Machine Learning Prediction

As described in section “Step 2: Predict Impairment via ML,” we use ML models to predict the impairment label, that is, a rat is impaired or not. Tables 2, 3 show the ML performance scores via different metrics as a result of implementing GNB, SVM, and ANN classifiers to predict impairment of ^56^Fe irradiated rats. The results for the ^4^He and ^28^Si irradiated rats can be found in Supplementary Tables 2, 3. Because 95% of sham and most irradiated rats are non-impaired, causing an imbalance between the positive class (impaired) and the negative class (non-impaired), we train the classifiers with class weighting as described in section “Cross-Validation, Class Weighting” to prevent a bias toward the majority class.

**TABLE 3:** Performance of classifiers when predicting impairment of sham and 10 cGy of ^56^Fe irradiated rats in the post-irradiation ATRC scores of the SD and CD stages.

Impairment threshold	ML algorithm	% Accuracy	AUC-ROC	AUC-PR	MCC	F_1_ score
SD ATRC ≥ 16	GNB	84	0.71	0.37	0.45	0.54
	SVM	83	0.71	0.60	0.38	0.48
	ANN	86	0.70	0.32	0.48	0.56
CD ATRC ≥ 36	GNB	79	0.59	0.22	0.29	0.39
	SVM	50	0.62	0.23	0.03	0.21
	ANN	79	0.55	0.25	0.29	0.39

*The input features are the dose (0 or 10 cGy) and the prescreen ATRC scores.*

Table 2 reflects classifying sham rats along with those irradiated with 1, 5, or 10 cGy of ^56^Fe; and Table 3 includes sham and only the 10 cGy ^56^Fe irradiated rats. The reason to prefer showing these cases is that based on Figures 5C, 6C, any dose of ^56^Fe produces higher ATRC scores than the sham, and an incremental effect is depicted in the height of the bar plots and box plots when rats are exposed to a higher dose, as also depicted in the percentage of impaired rats in Figures 9C, 10C. Therefore, we expect the classifiers to be able to perform better than random chance when presented with the sham and the rats irradiated with any dose of ^56^Fe, and to perform even better when asked to discriminate between the effect of 0 and 10 cGy of ^56^Fe than when presented with multiple doses of ^56^Fe.

Indeed, in each table, all classifiers have comparable success in predicting impairment exceeding a random guess; that is, we observe an accuracy score above 50%, an AUC-ROC above 0.5, an AUC-PR above the corresponding baseline of about 0.14, and a positive MCC. We also notice mostly higher numbers in Table 3 than in Table 2 as we anticipated. It is noteworthy to mention that SVM gives an MCC of almost 0 when classifying impairment in the CD stage and has the lowest F_1_ score. GNB and ANN perform better than SVM in the CD stage and almost identically to each other.

**FIGURE 11:**
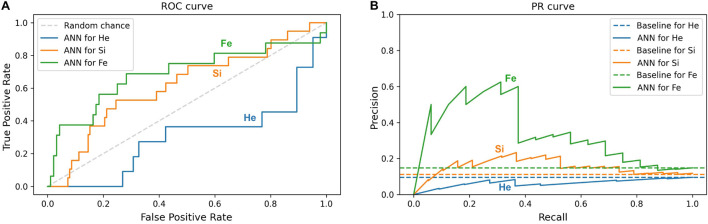
Impairment prediction in the SD stage using the ANN classifier. Each line corresponds to sham rats and those irradiated with ^4^He (blue), ^28^Si (orange), or ^56^Fe (green) ion at any of the given doses: 1, 5, and 10 cGy. **(A)** The ROC curve of the neural network. The dotted diagonal represents random chance. A “better-than-chance” prediction translates into an AUC above 0.5. **(B)** The PR curve of the neural network. Each dotted horizontal line represents the ratio of impaired rats in the corresponding group. A “better-than-chance” prediction translates into an AUC larger than the AUC of the corresponding baseline.

**FIGURE 12:**
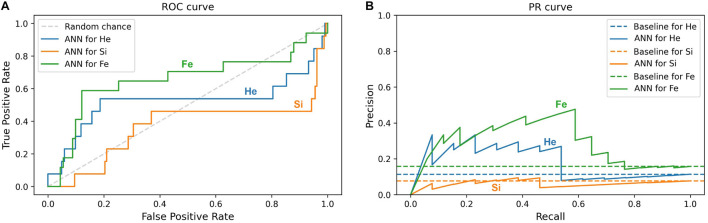
Impairment prediction in the CD stage using the ANN classifier. Each line corresponds to sham rats and those irradiated with ^4^He (blue), ^28^Si (orange), or ^56^Fe (green) ion at any of the given doses: 1, 5, and 10 cGy. **(A)** The ROC curve of the neural network. The dotted diagonal represents random chance. A “better-than-chance” prediction translates into an AUC above 0.5. **(B)** The PR curve of the neural network. Each dotted horizontal line represents the ratio of impaired rats in the corresponding group. A “better-than-chance” prediction translates into an AUC above the AUC of the corresponding baseline.

Furthermore, this study compares the ML prediction for the ^4^He, ^28^Si, and ^56^Fe ions and checks if the prediction is better than random chance for all ions. An efficient way to perform and visualize this comparison is by plotting the ROC and PR curves (Micheal, 1997; Fawcett, 2004; Tharwat, 2018). A classifier is a better predictor when it reveals a larger AUC-ROC, and its ROC curve is closer to the upper left corner of ROC space, and when the AUC-PR is larger, and the corresponding PR plot is closer to the upper right corner in PR space. Impairment prediction via ANN is compared in Figure 11 for the SD stage and in Figure 12 for the CD stage. For each ion, the corresponding curves include sham rats and those irradiated with 1, 5, or 10 cGy of the designated ion.

## Discussion

The main objective of this paper is to predict rodent performance impairment in stages of the ATSET at a subject level after single GCR ion irradiation. Traditional population-based analyses, such as the Mann–Whitney test, show the presence of cognitive deficits as a trend at a group level, but are insufficient for our purpose to individualize the effect. We utilize statistical tools, i.e., the CDF, to define the cognitive impairment threshold by analyzing the post-irradiation scores of the shams’ cohort at the group level. We then employ ML algorithms to pinpoint the impaired rats from their groups. Therefore, as discussed in this section, ML models supplement population-based statistical analyses through their strong predictive capabilities to extend the results to a subject-based level.

### Classifier Performance

An interesting observation from the application of ML techniques to the prediction of cognitive impairment relates to the need to utilize the prescreen scores as an additional feature to enhance classification. In the absence of the prescreen scores, the classifiers in this study rely exclusively upon the dose as input feature and would predict all rats exposed to one dose to be either impaired or not based on the majority class. Since most rats are non-impaired regardless of the radiation exposure level, as seen in Figures 9, 10, then the ML model would not be able to single out any impaired rat. Results from the inclusion of prescreen scores as additional input features indicate that the ability to predict impairment is potentially dependent on the individual measured cognitive capability of rat subjects before and after irradiation.

Indeed all scores in Tables 2, 3 reflect better-than-chance prediction capability when considering sham and ^56^Fe irradiated rats, as shown in section “Machine Learning Prediction,” with an accuracy score above 50%, an AUC-ROC above 0.5, an AUC-PR above the approximate baseline of 0.14, and a positive MCC. A better-than-chance prediction shows that the model is capable of establishing a relation between the input features and the impairment. In particular, because Table 2 considers sham rats and those exposed to 1, 5, or 10 cGy of ^56^Fe ions and shows better-than-chance predictive capability in both SD and CD stages, then we can predict and ascertain the presence of dose-effect for ^56^Fe, as also depicted in the percentage impaired rats at different doses in Figures 9C, 10C. We discuss this dose-effect further in section “Percent of Impaired Subjects.” Here we mention that experimental studies show cognitive deficits in rodents exposed to ^56^Fe irradiation at doses as low as 20 cGy in the Barnes maze (Britten et al., 2012) and 10 cGy in the novel object recognition test (Cherry et al., 2012; Patel et al., 2017).

In Figures 11A,B involving the SD stage, the ROC and PR curves of the ANN prediction reveal AUC-ROC of 0.39, 0.62, and 0.69 for ^4^He, ^28^Si, and ^56^Fe, respectively, and AUC-PR of 0.08, 0.16, and 0.37, respectively. These numbers show that the ANN is more capable of distinguishing SD impairment after irradiation with ^56^Fe than after exposure to ^28^Si and is less robust in the ^4^He ion case where the AUC-ROC and AUC-PR are both below their “random chance” scores of 0.5 and 0.13, respectively. The ability of a classifier to distinguish impairment indicates that it can establish a relation between the dose and prescreen scores on the one hand and the impairment label on the other. Similarly, in Figures 12A,B for the CD stage, the AUC-ROC are 0.53, 0.35, and 0.66 for ^4^He, ^28^Si, and ^56^Fe, respectively, and the AUC-PR are 0.18, 0.06, and 0.32. These numbers show that the ANN can distinguish CD impairment after irradiation with ^56^Fe more than ^4^He and is limited in its predictive ability in the ^28^Si case. The relevance of this result is that we can identify which rats are at a higher risk of impairment if such risk exists. Moreover, traditional statistical methods already allude to the presence of such risk at a group level: the average ATRC CD score of a 10 cGy ^4^He or ^56^Fe irradiated rat is higher than that of a rat exposed to 10 cGy of ^28^Si as seen in Figures 5A–C, and the percentage of irradiated rats in that stage is higher for ^4^He and ^56^Fe than it is for ^28^Si as seen in Figures 10A–C.

### Percent of Impaired Subjects

To further understand the difference among ions in the prediction capability of the ANN, we inspect the percentage of rats that are impaired for different ions and different doses plotted in Figure 9 for the SD stage and Figure 10 for CD. In Figures 9B,C, the higher the dose of irradiation with ^28^Si or ^56^Fe ions, the higher the percentage of impaired rats in SD, with a more distinctive response for ^56^Fe. This indicates a dose-response in the SD stage, when rats are irradiated with ^28^Si or ^56^Fe ions. It is presumed that the SD stage is mainly regulated by the medial prefrontal cortex (Jewell et al., 2018), one of the regions involved in the novel object recognition test, in which literature shows deficit after irradiation with ^28^Si or ^56^Fe ions (Rabin et al., 2014; Patel et al., 2017).

We note that the Mann–Whitney test does not find a statistically significant difference between the sham cohort and the 1 or 5 cGy ^56^Fe irradiated rats in the SD stage as seen in Figure 5C, although the ML algorithms and the cumulative distribution plots are able to depict some severely impaired rats in those groups. This is because the statistically significant difference reports how each irradiated group is different than the sham cohort when comparing their population medians, whereas our approach is interested in individuals’ impairment. Section “Cumulative Distributions and Impairment Thresholds” gives an SD threshold score of 16, and looking at Figure 6C the impaired rats are outliers, far from the median. Therefore, when our results indicate a dose-effect, if it seems contradictory to the results from traditional statistical methods, keep in mind that we are investigating the cognitive impairment or extreme deficits, not just the low cognitive performance.

Figure 10C shows the dose-effect from exposure to ^56^Fe in the CD stage, where rats irradiated with any dose are at least four times more susceptible to impairment than sham rats. Additionally, Figure 6C shows that the higher the ^56^Fe ion dose, the more the variability among subjects as seen from the interquartile range increasing with the dose. We hypothesize that an ^56^Fe radiation dose of less than 1 cGy causes deficits in the CD stage. A prominent effect at 10 cGy of ^4^He ion is detected, suggesting a dose in the range of 5–10 cGy of ^4^He is responsible for inducing cognitive deficit in the brain regions and pathways involved in the CD stage. For the ^28^Si ion, we notice in Figure 10B that 5 cGy shows a similar effect to that of no radiation at all, whereas 1 and 10 cGy show a higher number of impaired rats, which leads to more difficulty for the classifier to relate impairment to radiation. This is seen in the low-performance metrics of the ANN, as detailed in Supplementary Table 2. Further testing with more rodent subjects would be beneficial to clarify this non-monotonic dose-response to ^28^Si irradiation.

The literature suggests that SD and CD are regulated by different parts of the brain (Jewell et al., 2018; Britten et al., 2021), potentially justifying the disparity between the classification prediction capability in SD and CD performance for different ions. Particularly, Figures 11, 12 suggest that ^56^Fe or ^28^Si irradiation causes damage in areas of the brain regulating the SD stage, while ^56^Fe or ^4^He irradiation affects the parts regulating the CD stage. However, the damage by ^4^He (or ^28^Si) irradiation may involve other aspects of cognitive damage not quantified by SD (or CD, respectively), or ^4^He (or ^28^Si) ion is not as deleterious as the other two ions. The fact that ^56^Fe irradiation affects both the SD and CD stages suggests that multiple areas or pathways in the brain are affected by the irradiation.

We comment in section “Cumulative Distributions and Impairment Thresholds” about the cumulative distribution MCL not showing a higher percentage of impaired rats for most irradiated groups. The ML classifiers are thereby unable to predict MCL impairment. This suggests that the time spent by a rodent subject to retrieve a food reward correctly does not necessarily increase even if their ATRC increases when irradiated. This is not surprising as the MCL bar plots in Supplementary Figure 1 show no significant difference between the sham and various groups irradiated with ^4^He, ^28^Si, or ^56^Fe ions.

### Limitations

Although the results from our ML models in Figures 11, 12 and Tables 2, 3 are better than random chance, one might wonder why they are this low. In other words, why do the ML models result in so many misclassifications if they are well trained? We inspect our dataset samples closely and observe many cases where two rats having similar or even equal scores in the prescreen test and being irradiated with the same dose of an ion result in different impairment labels. Such cases are also seen in the sham group. An ML model treats these two samples the same and predicts the same impairment label, leading to one misclassification. With our dataset of low sample size, these discrepancies heavily affect ML performance and result in more classification errors.

It may be possible to get higher ML performance scores if one implements a more systematic approach and takes time to tune the algorithm parameters for optimization. However, that would not change the key conclusions of this study, related to the better-than-random-chance prediction capability.

Like most data in the effect of GCR radiation research, the data used in this study is limited by the low sample size (Table 1). This leads to a higher error range when performing population analyses and requires additional steps of bootstrapping and cross-validation in ML to take advantage of all possible data points (see section “Cross-Validation, Class Weighting”).

Another limitation in the data on hand is reproducibility, a recurrent topic of discussion in the health and biomedical sciences research community. Data used in our study include lab runs that occurred from years 2014 to 2018. This implies many changes in human and animal involvement, such as including multiple cohorts of rats though with the same specs and employing different animal handlers. As minimal as they are kept, these unavoidable changes can still affect behavioral testing and scoring, as is often addressed in rodents psychology literature (Gulinello et al., 2018; Kafkafi et al., 2018). This limitation will be more pronounced when validating our results with data from other experimental studies.

Behavioral studies have shown high inter- and intra-individual variabilities in animals and humans in response to brain stimulation paradigms (Fiske and Rice, 1955; Loevinger, 1957; López-Alonso et al., 2014; Wyrobek and Britten, 2016; Sherman and Sladky, 2018). These variabilities are manifested when the same subject responds differently in the ATSET if the test is done another time and may provide a potential explanation for rats with similar prescreen scores having different impairment labels. Further variations arise due to the use of the outbred Wistar rats (Wyrobek and Britten, 2016).

There may be other factors contributing to the performance change after irradiation that are not captured in the current datasets. More behavioral tests and biomarkers should be investigated on the same subjects. Additional pertinent variables about each rodent subject, such as daily vital signs and exact time of the day when the assay was administered, should be recorded during the experiments even if not deemed necessarily useful at the time. These variables can be fed to the ML models to capture the agents responsible for performance alterations of some individuals versus others. It is advisable to capture video recordings of the rodents. With the advancement of ML algorithms and artificial intelligence and the growing computer power, more tools are available every day to glean from data that the human brain cannot readily extract. Those tools can use the additional variables and test recordings provided by lab technicians to unravel potential hidden relationships between dose exposure and cognitive impairment.

One should be cautious in extrapolating any results from a single-ion beam exposure experiment directly to spaceflight, as astronauts will be exposed simultaneously to a variety of ions during deep space missions. Building models to combine the effects of single-ion exposure is an ongoing area of investigation. NSRL has recently built a simplified five-ion mixed field beam that simulates the top five contributors to GCR radiation (Simonsen et al., 2020). Researchers now have the opportunity to use the facility to irradiate rodents with a mixed-beam and use the data to test and validate their models for predicting combined effects from single-beam exposure (Pani et al., 2016; Huang et al., 2019). In addition, microgravity and isolation/confinement are also space factors that can potentially affect the cognitive performance of astronauts. Incorporating the simultaneous effects of these stressors with radiation is another active research area (Kokhan et al., 2019; Chappell et al., 2020; Willey et al., 2021).

### Outlook

The ML algorithms that we employ in this study hold different mathematical backgrounds, i.e., GNB is probabilistic, SVM is geometric, and ANN is network-based. Each one of them has its limitations but can still predict better than chance. For example, GNB assumes that the input features are independent, SVM that they are linearly separable, and ANN is computationally expensive. With more data available, a future direction could be to build an ML ensemble method to take advantage of the strengths of each classifier for a more robust and generalized computational framework.

In investigating the effects of GCR on rodents, there is limited behavioral and physiological data, driving impairment predictions on a group level rather than subject level, assuming that all individuals in a group have the same susceptibility to impairment. For an impairment prediction tailored to each subject, we recommend administering more animal studies with a larger sample size and work toward understanding the translation of the results to humans. This is imperative given the severity of potential effects of astronauts’ cognitive deficits on mission success and safety.

## Data Availability Statement

The data analyzed in this study is subject to the following licenses/restrictions: US NASA NNX14AE73G. Requests to access these datasets should be directed to RB, brittera@evms.edu.

## Author Contributions

MM procured, cleaned, and preprocessed the data in the database, guided the technical direction of the study, contributed to discussions, performed the population statistical analyses including impairment thresholds and percentages, implemented the Artificial Neural Network algorithm, created all visualizations in the manuscript, and composed the first draft of the manuscript. SG managed conceptualization and technical task coordination, led discussions, guided the technical direction of the study, and provided critical peer review and interpretation of the results. MP implemented the Support Vector Machine algorithm and contributed to discussions. CG implemented the Support Vector Machine algorithm. AI implemented the Gaussian Naïve Bayes algorithm. RB provided the data from own previous experiments and helped in the interpretation of the experiment and data. RP coordinated the project, led discussions, guided the technical direction of the study, and provided critical peer review and interpretation of the results. JM coordinated the project, ensured the implementation of the model credibility criteria, led discussions, guided the technical direction of the study, and provided critical peer review and interpretation of the results. All authors contributed to manuscript revisions, read, and approved the submitted version.

## Conflict of Interest

During the time of conducting the submitted study, author AI was employed by ZIN Technologies, and author RP was employed by USRA. AI and RP were employees of contractors funded for this work by NASA’s Human Research Program. The remaining authors declare that the research was conducted in the absence of any commercial or financial relationships that could be construed as a potential conflict of interest.

## Publisher’s Note

All claims expressed in this article are solely those of the authors and do not necessarily represent those of their affiliated organizations, or those of the publisher, the editors and the reviewers. Any product that may be evaluated in this article, or claim that may be made by its manufacturer, is not guaranteed or endorsed by the publisher.

## Footnotes

1 POLs: Acceptable maximum decrement or change in a physiological or behavioral parameter, during or after a space flight mission, as the result of exposure to the space environment.

2 SPELs: Quantifiable limit of exposure to a space flight factor over a given length of time.
