# Supplementary material for: Machine Learning Models to Predict Cognitive Impairment of Rodents Subjected to Space Radiation
Source: Front Syst Neurosci. 2021 Sep 13;15:713131. doi: 10.3389/fnsys.2021.713131 (PMC8473791; doi:10.3389/fnsys.2021.713131)
Supplement: Supplementary file 1 [file Data_Sheet_1.docx]

Supplementary Materials

**SUPPLEMENTARY FIGURES**


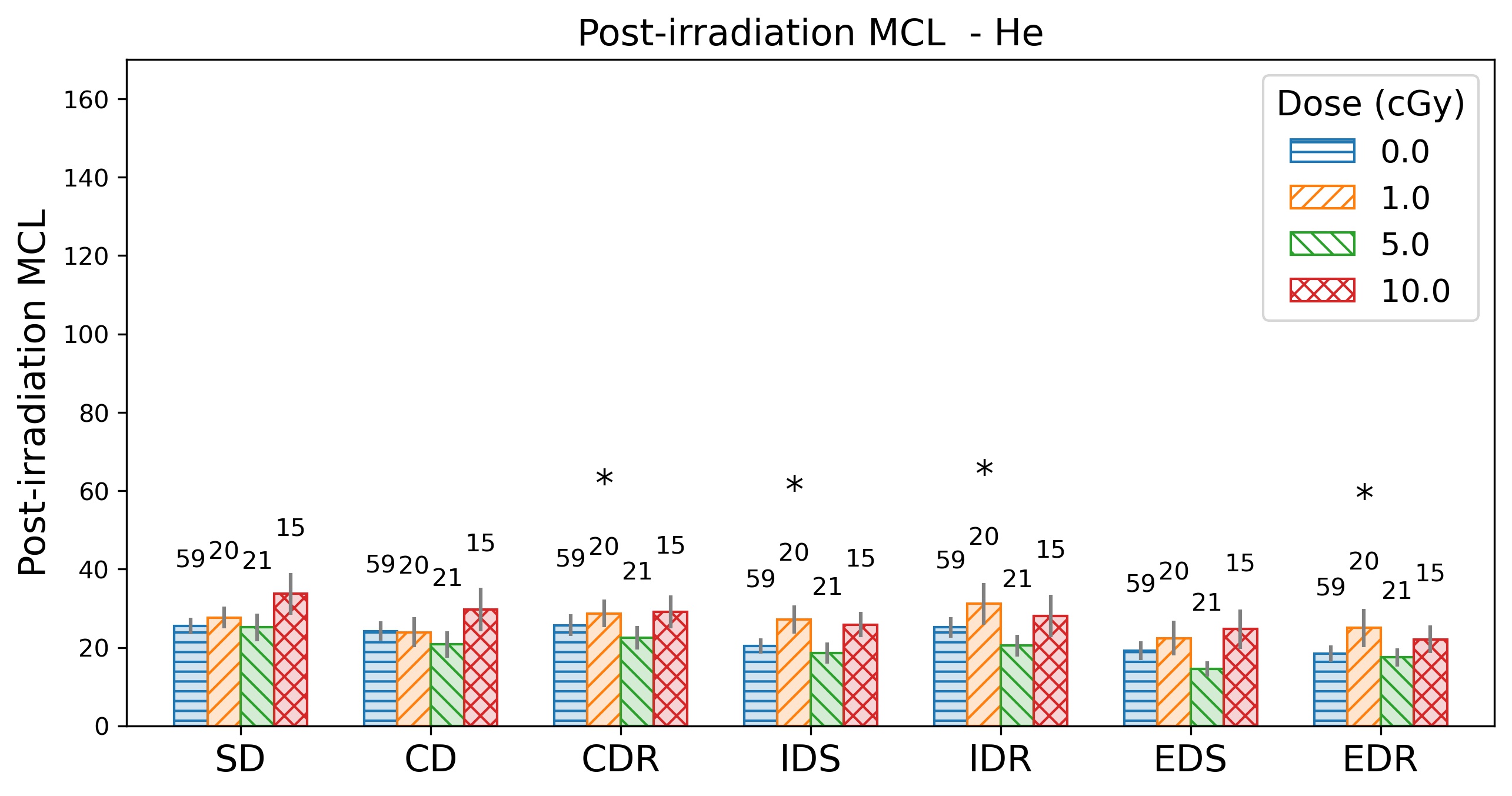


**A**


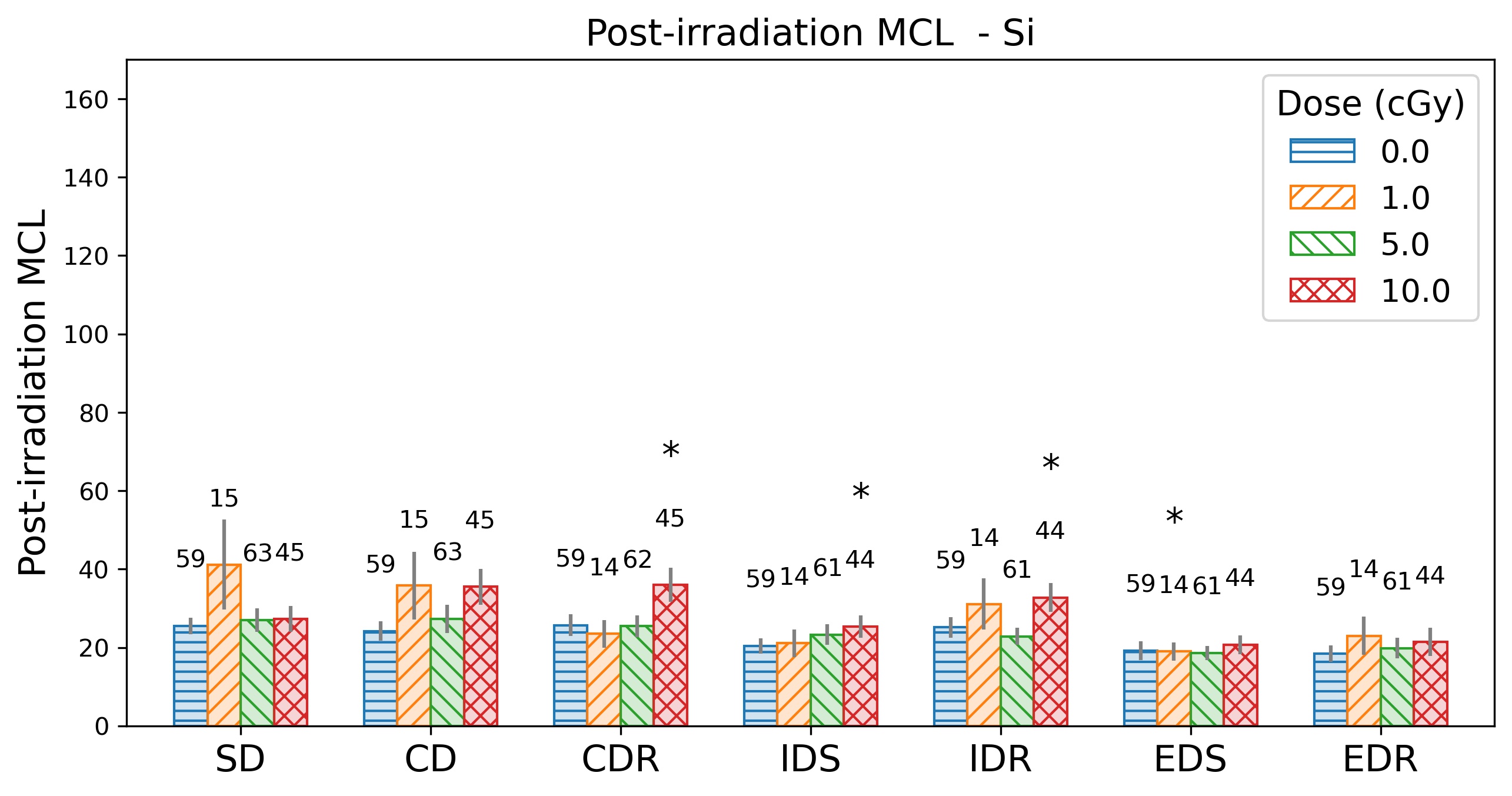


**B**


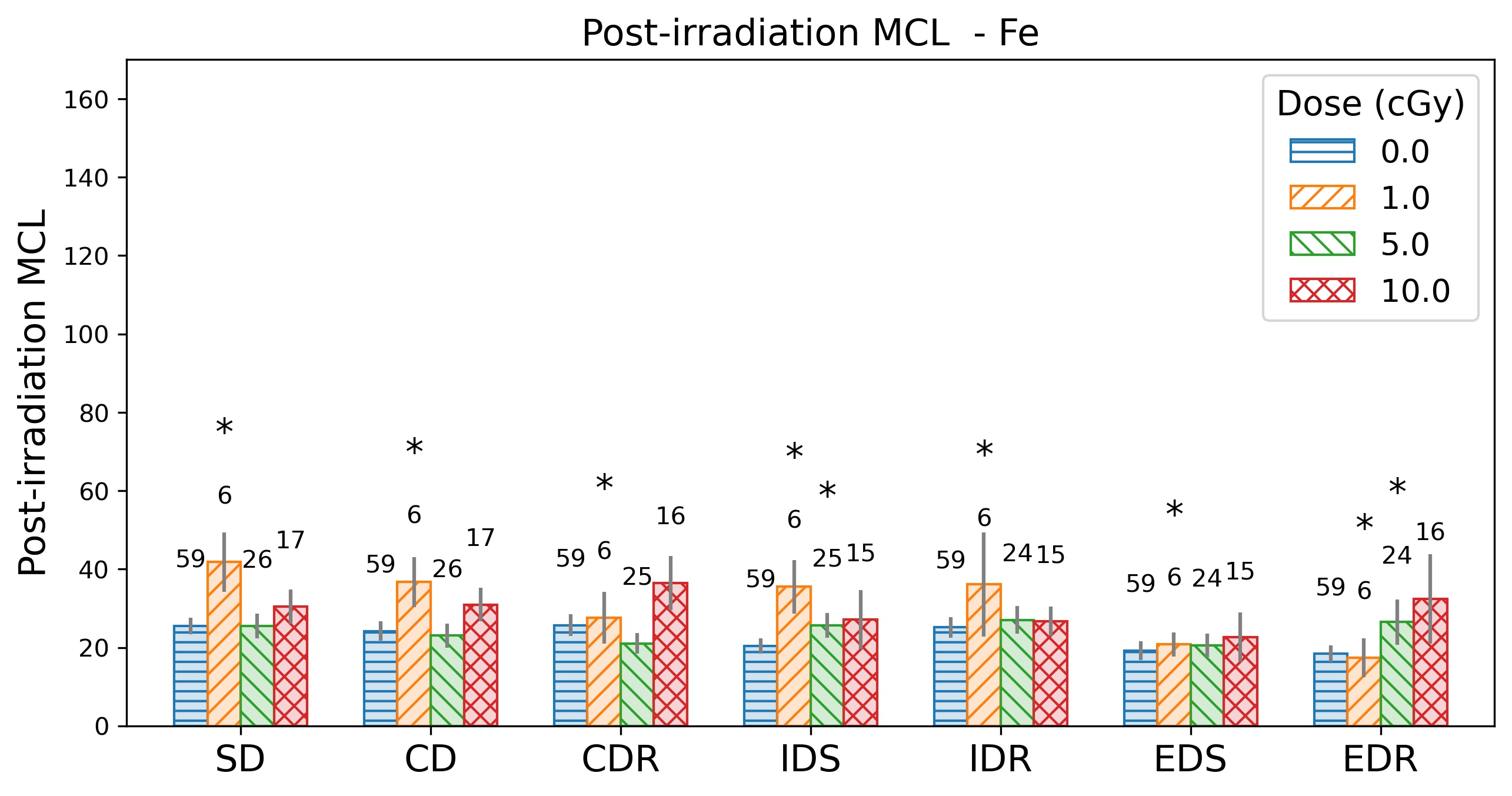


**C**

**Supplementary Figure 1:** Bar plots showing the mean and SEM of MCL scores for different ATSET stages post-irradiation with (A) ^4^He, (B) ^28^Si, or (C) ^56^Fe ions. The numbers above the bars are the sample size. Asterisks refer to groups that are significantly different from the sham group using the Mann-Whitney test.


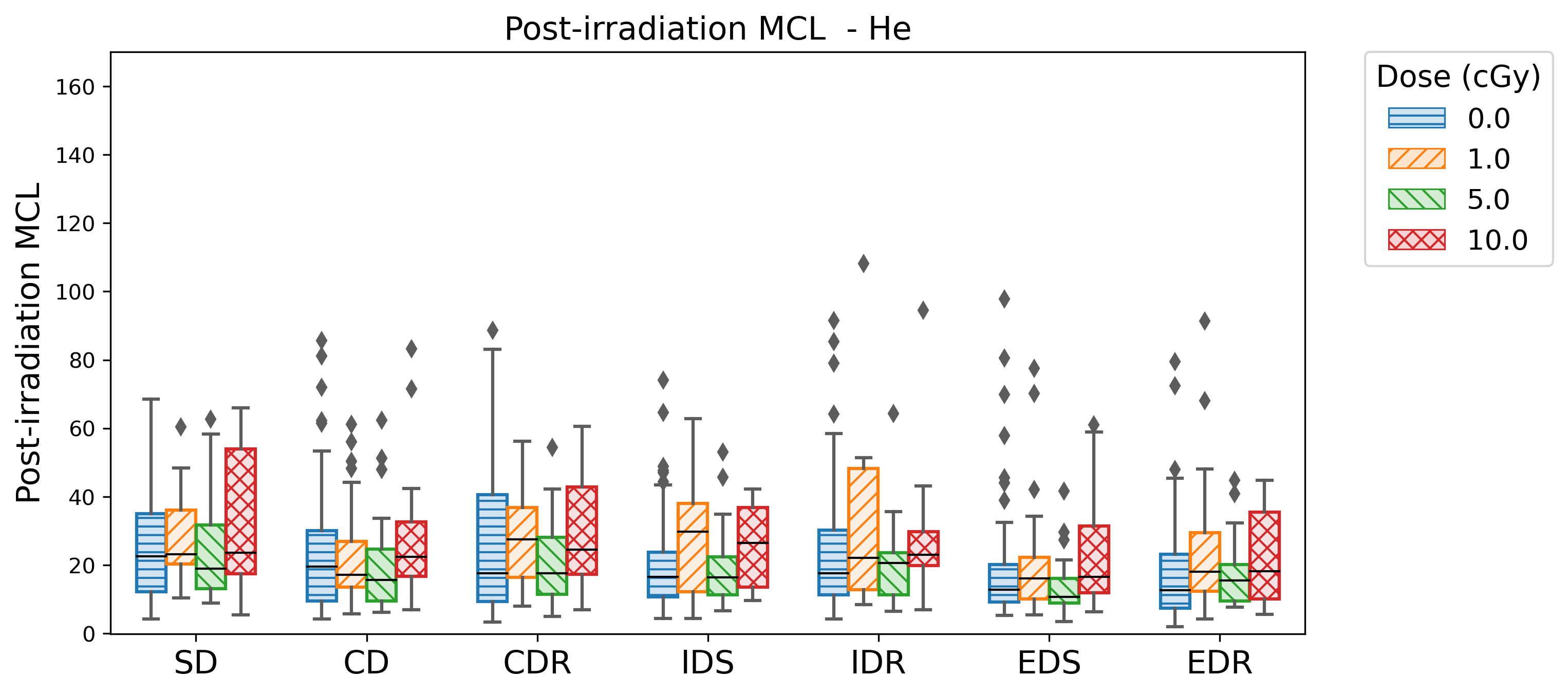


**A**


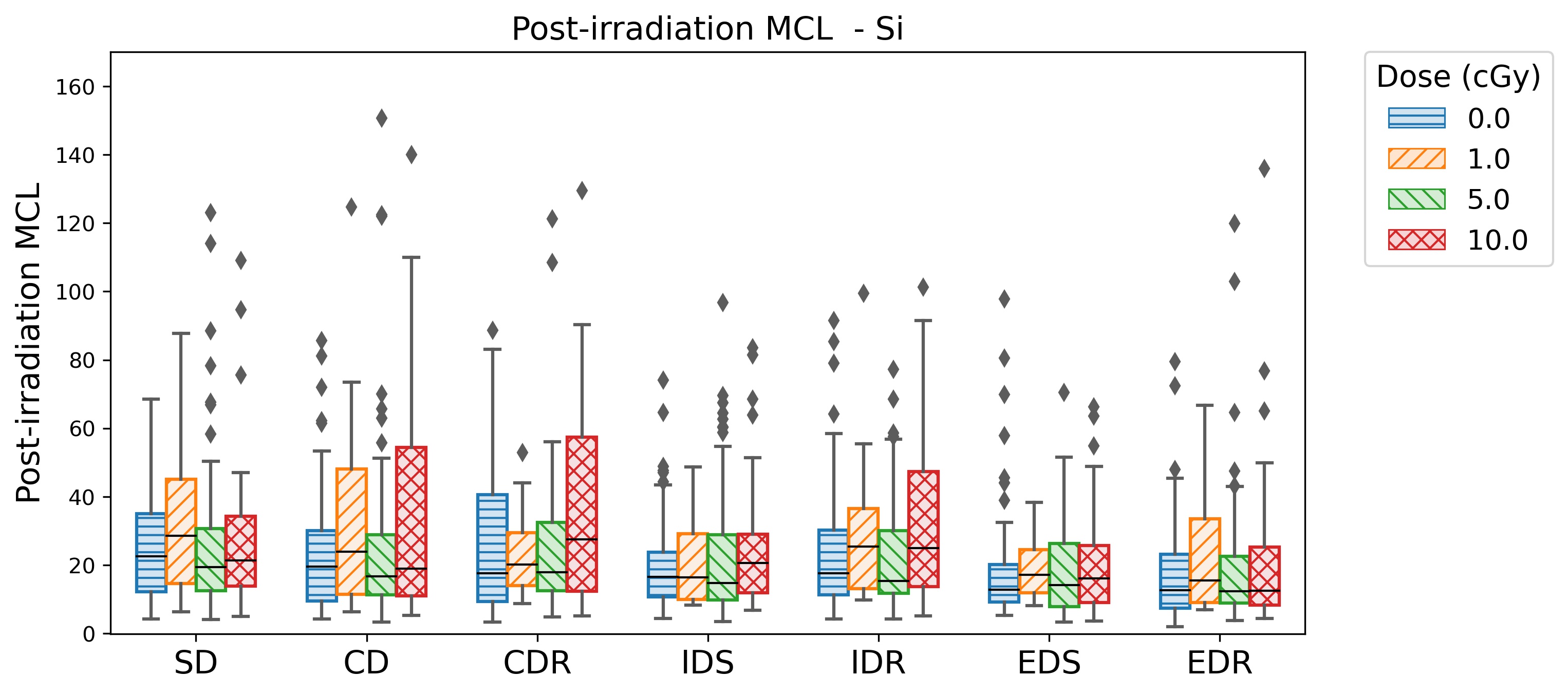


**B**


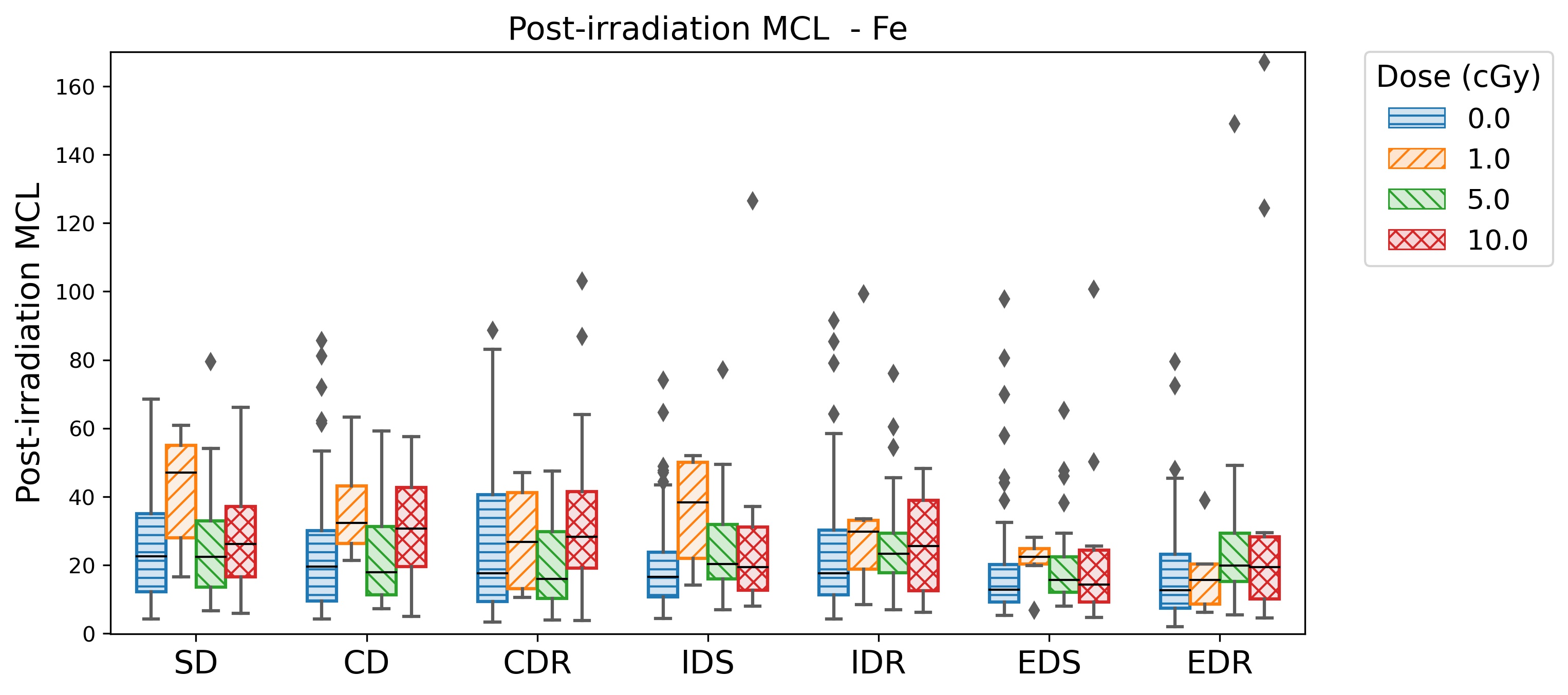


**C**

**Supplementary Figure 2:** Box plots showing the median, quartiles, and outliers of MCL scores for different ATSET stages post-irradiation with (A) ^4^He, (B) ^28^Si, or (C) ^56^Fe ions.


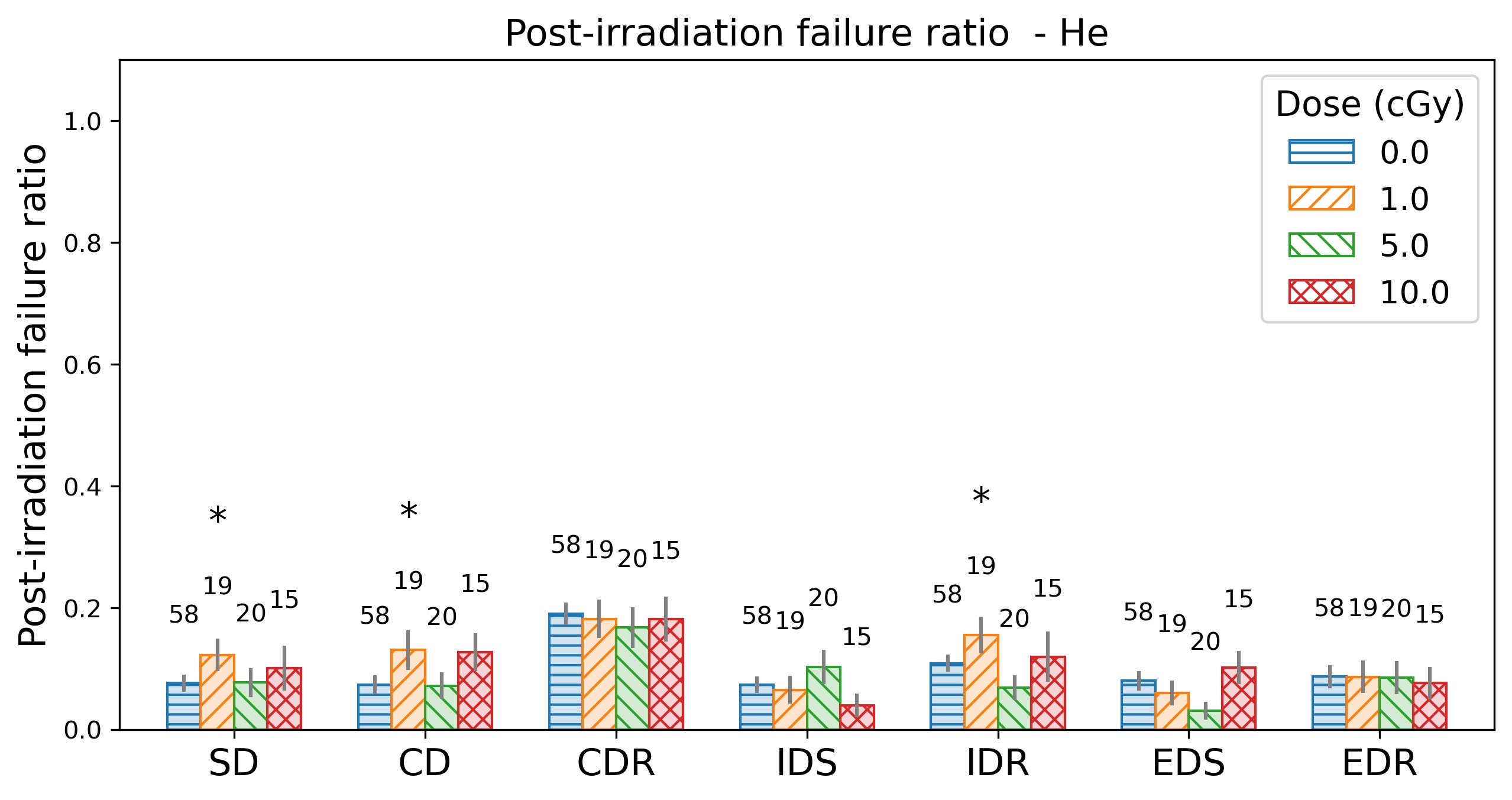


**A**


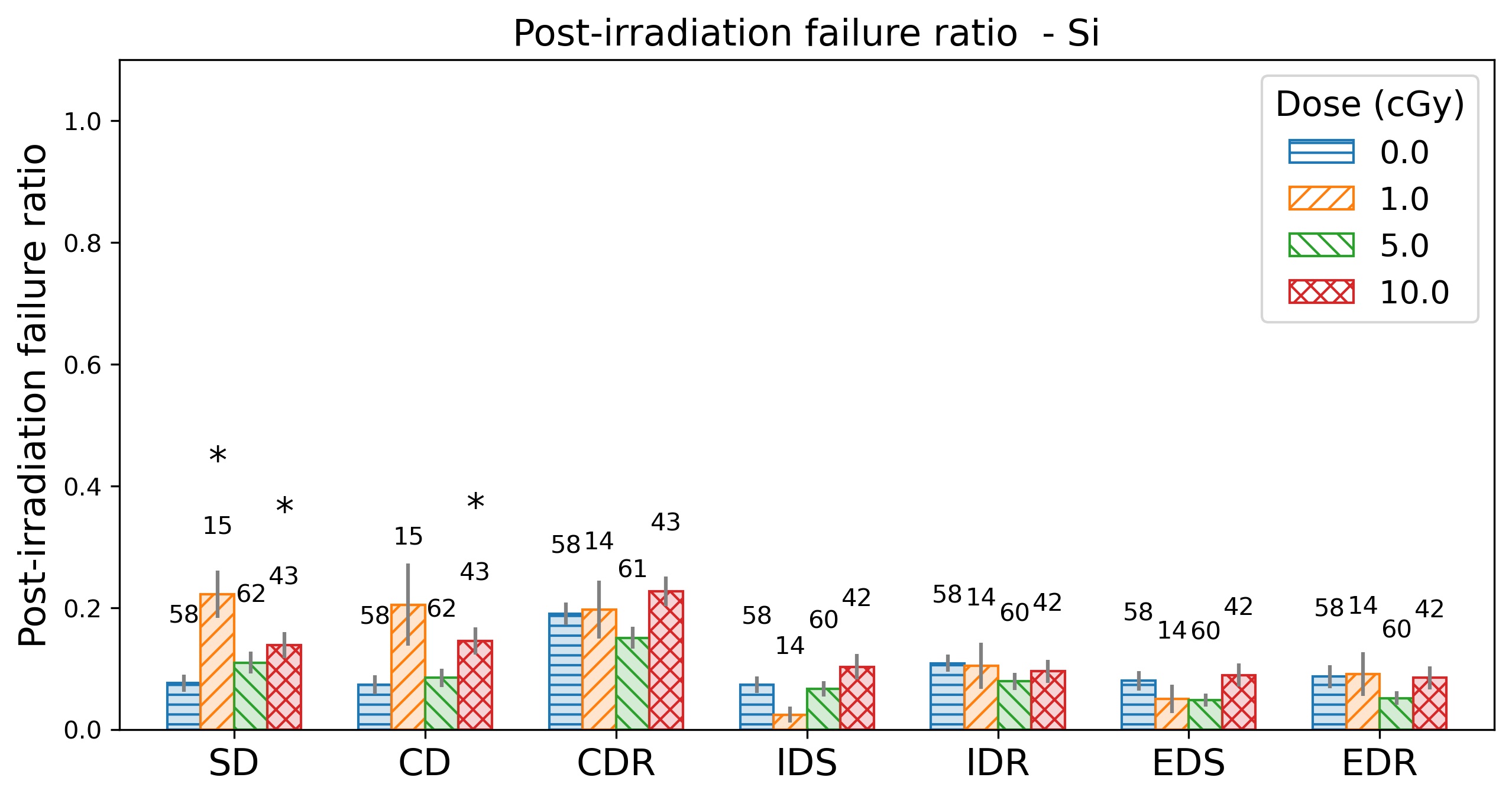


**B**


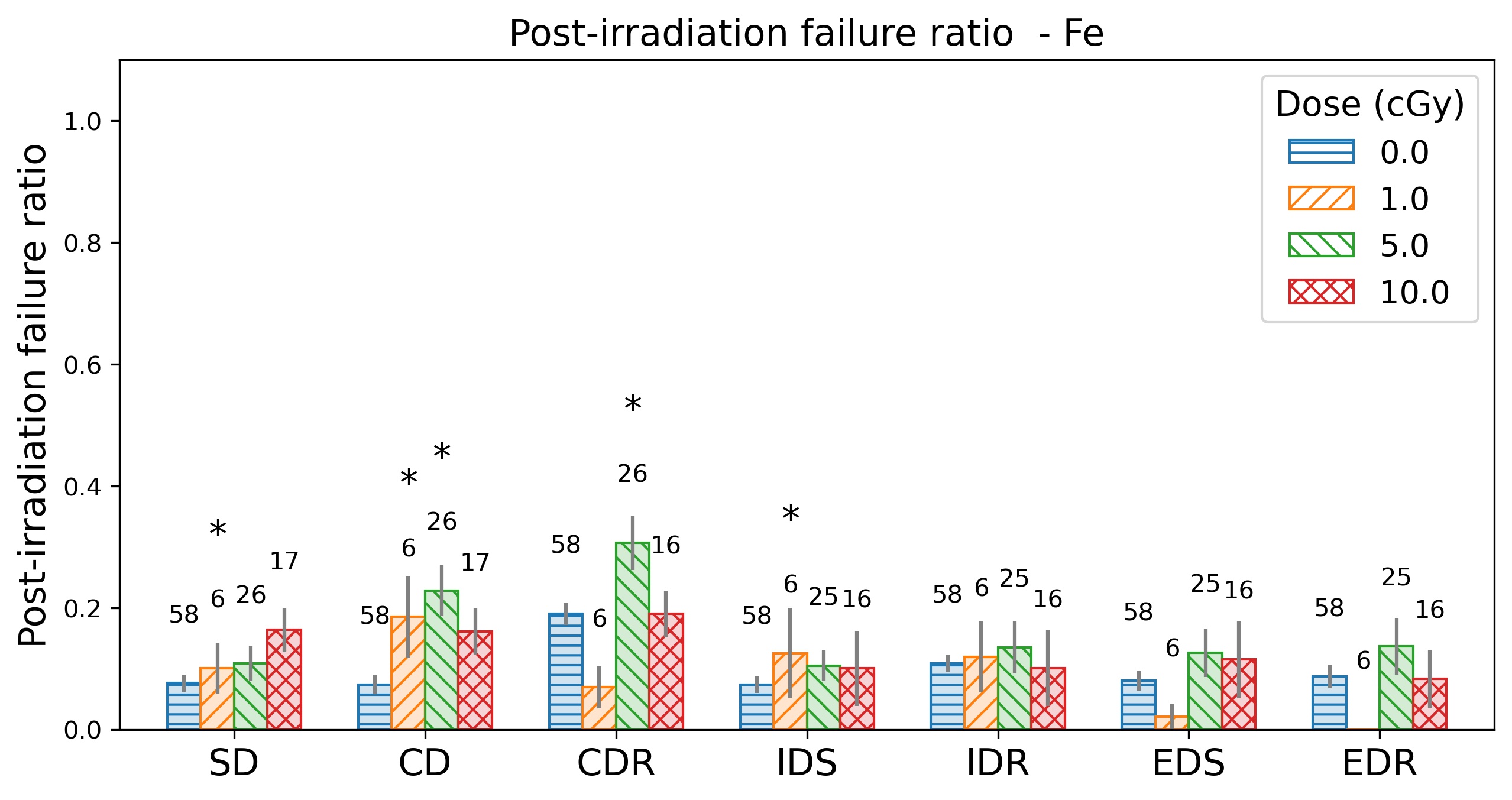


**C**

**Supplementary Figure 3:** Bar plots showing the mean and SEM of FR scores for different ATSET stages post-irradiation with (A) ^4^He, (B) ^28^Si, or (C) ^56^Fe ions. The numbers above the bars are the sample size. Asterisks refer to groups that are significantly different from the sham group using the Mann-Whitney test.


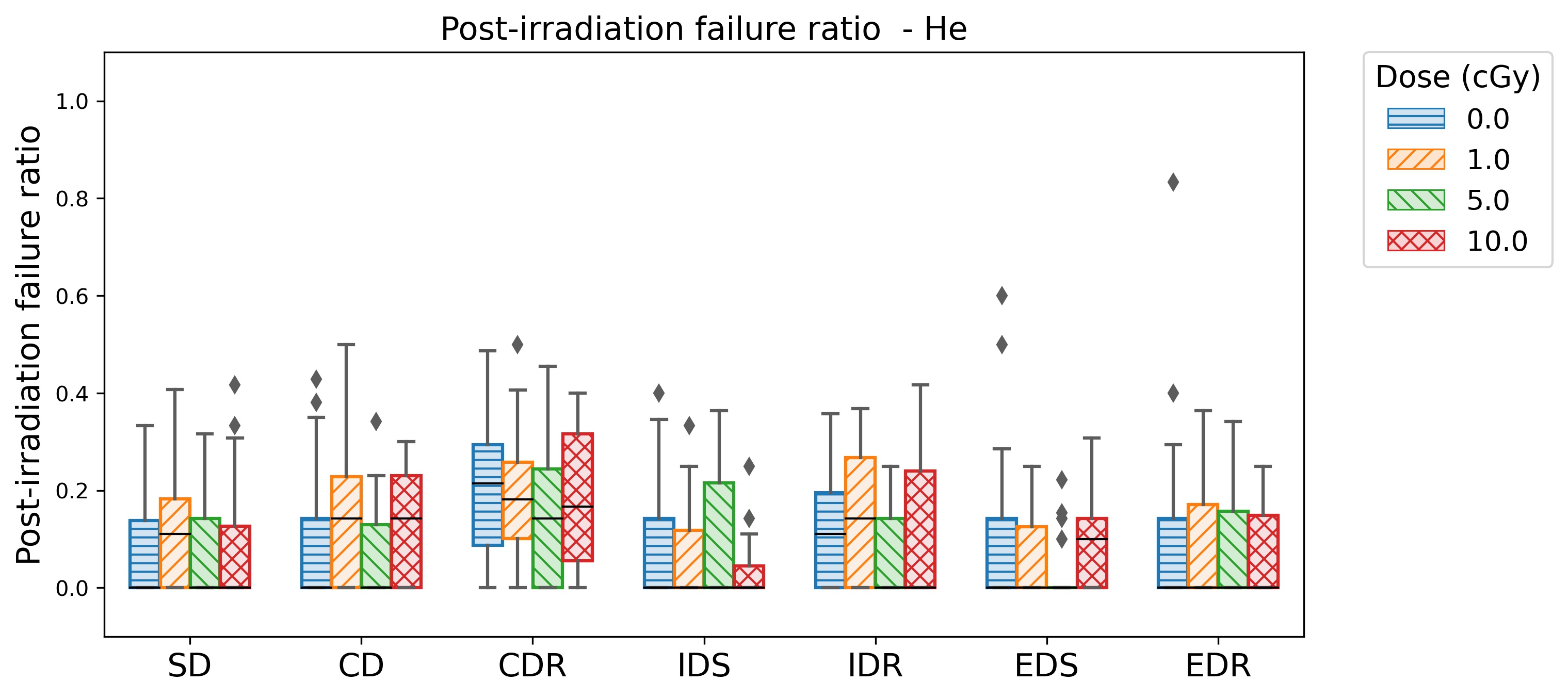


**A**


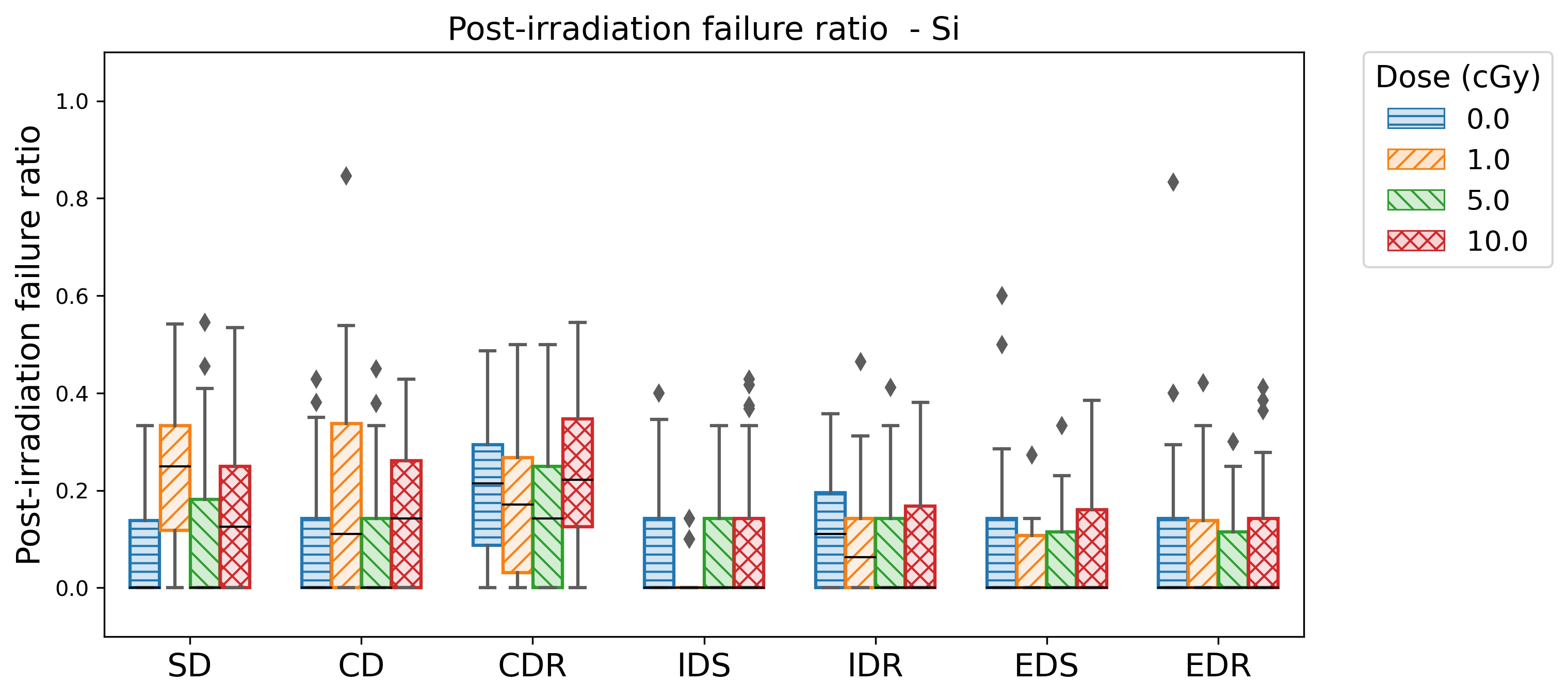


**C**

**B**


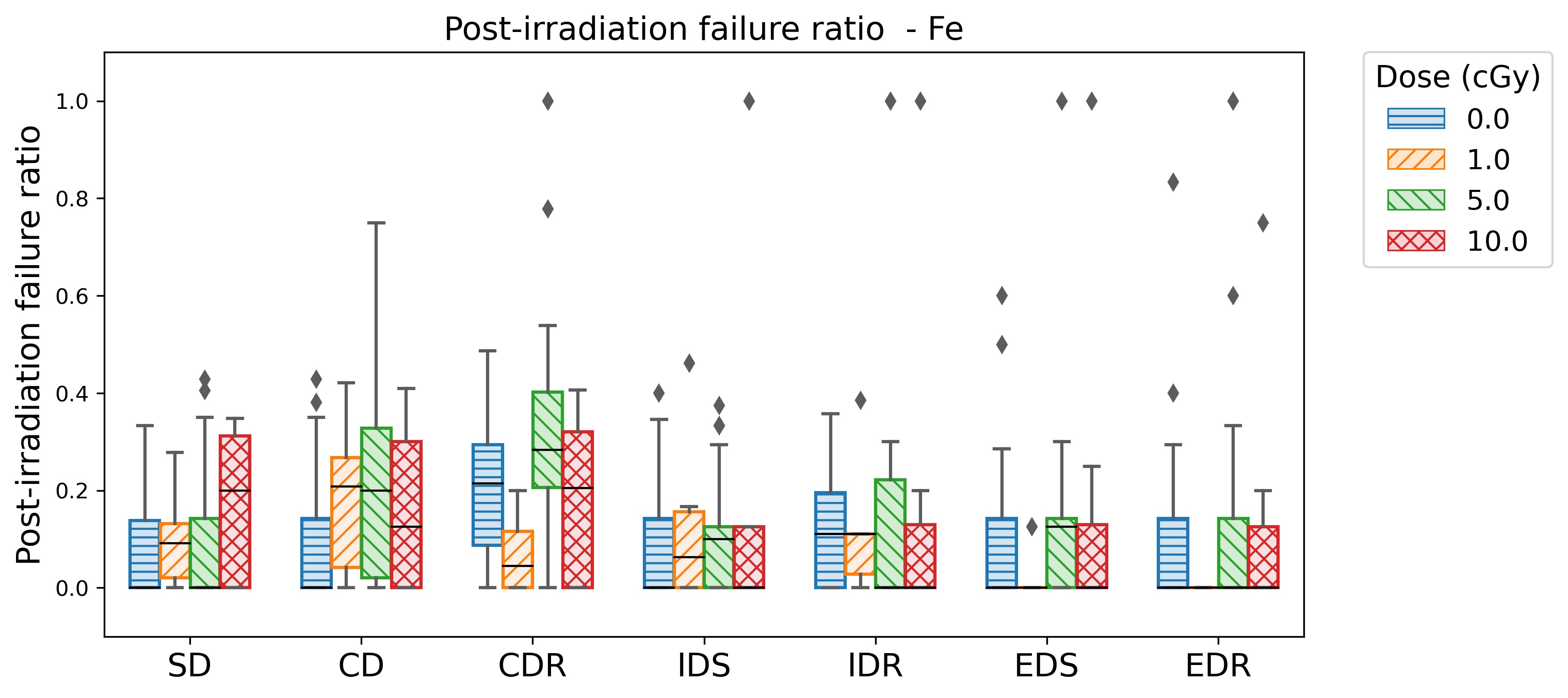


**Supplementary Figure 4:** Box plots showing the median, quartiles, and outliers of FR scores for different ATSET stages post-irradiation with (A) ^4^He, (B) ^28^Si, or (C) ^56^Fe ions.

**SUPPLEMENTARY TABLES**

***Supplementary Table 1:*** Impairment thresholds in ATRC and MCL scores in all 7 stages of the ATSET. These threholds are determined by considering the 15% worst performing sham rats as impaired (85% Threshold), the worst 10% (80% Threshold), and the worst 5% (95% Threshold).

| **ATSET Score and Stage** | **85% Threshold** | **90% Threshold** | **95% Threshold** |
| --- | --- | --- | --- |
| **ATRC SD** | 12 | 13 | 16 |
| **ATRC CD** | 13 | 20 | 36 |
| **ATRC CDR** | 41 | 42 | 44 |
| **ATRC IDS** | 11 | 13 | 17 |
| **ATRC IDR** | 12 | 14 | 19 |
| **ATRC EDS** | 12 | 14 | 22 |
| **ATRC EDR** | 13 | 14 | 15 |
| **MCL SD** | 47 | 51 | 53 |
| **MCL CD** | 42 | 53 | 72 |
| **MCL CDR** | 49 | 55 | 79 |
| **MCL IDS** | 36 | 44 | 49 |
| **MCL IDR** | 47 | 54 | 79 |
| **MCL EDS** | 28 | 44 | 70 |
| **MCL EDR** | 34 | 43 | 48 |

**Supplementary Table 2:** Performance of classifiers when predicting impairment of sham and He, Si, or Fe irradiated rats in the post-irradiation ATRC scores of the SD and CD stages. The input features are the dose (0, 1, 5, or 10 cGy) and the prescreen ATRC scores.

| Ion | Impairment threshold | ML algorithm | % Accuracy | AUC-ROC | AUC-PR | MCC | F_1_ score |
| --- | --- | --- | --- | --- | --- | --- | --- |
| He | SD ATRC ≥ 16 | GNB | 44 | 0.29 | 0.07 | -0.25 | 0.03 |
|  |  | SVM | 77 | 0.56 | 0.14 | -0.06 | 0.07 |
|  |  | ANN | 63 | 0.39 | 0.08 | -0.04 | 0.12 |
|  | CD ATRC ≥ 36 | GNB | 70 | 0.61 | 0.20 | 0.17 | 0.29 |
|  |  | SVM | 88 | 0.52 | 0.33 | 0.31 | 0.36 |
|  |  | ANN | 76 | 0.53 | 0.26 | 0.24 | 0.33 |
| Si | SD ATRC ≥ 16 | GNB | 58 | 0.61 | 0.21 | 0.10 | 0.23 |
|  |  | SVM | 77 | 0.55 | 0.17 | 0.12 | 0.24 |
|  |  | ANN | 62 | 0.62 | 0.16 | 0.13 | 0.26 |
|  | CD ATRC ≥ 36 | GNB | 62 | 0.43 | 0.08 | -0.10 | 0.06 |
|  |  | SVM | 86 | 0.35 | 0.07 | -0.07 | 0.00 |
|  |  | ANN | 68 | 0.37 | 0.07 | 0.01 | 0.13 |
| Fe | SD ATRC ≥ 16 | GNB | 66 | 0.68 | 0.33 | 0.21 | 0.35 |
|  |  | SVM | 81 | 0.65 | 0.37 | 0.30 | 0.41 |
|  |  | ANN | 71 | 0.69 | 0.37 | 0.25 | 0.38 |
|  | CD ATRC ≥ 36 | GNB | 72 | 0.64 | 0.28 | 0.30 | 0.42 |
|  |  | SVM | 74 | 0.58 | 0.30 | 0.07 | 0.22 |
|  |  | ANN | 75 | 0.66 | 0.32 | 0.30 | 0.43 |

**Supplementary Table 3:** Performance of classifiers when predicting impairment of sham and 10 cGy of He, Si, or Fe irradiated rats in the post-irradiation ATRC scores of the SD and CD stages. The input features are the dose (0 or 10 cGy) and the prescreen ATRC scores.

| Ion | Impairment threshold | ML algorithm | % Accuracy | AUC-ROC | AUC-PR | MCC | F_1_ score |
| --- | --- | --- | --- | --- | --- | --- | --- |
| He | SD ≥ 16 | GNB | 58 | 0.38 | 0.07 | -0.21 | 0.00 |
|  |  | SVM | 82 | 0.54 | 0.14 | 0.14 | 0.22 |
|  |  | ANN | 74 | 0.50 | 0.11 | 0.16 | 0.23 |
|  | CD ≥ 36 | GNB | 79 | 0.59 | 0.22 | 0.29 | 0.39 |
|  |  | SVM | 46 | 0.55 | 0.21 | 0.00 | 0.20 |
|  |  | ANN | 82 | 0.52 | 0.21 | 0.33 | 0.42 |
| Si | SD ≥ 16 | GNB | 63 | 0.53 | 0.14 | 0.15 | 0.28 |
|  |  | SVM | 70 | 0.60 | 0.21 | 0.16 | 0.29 |
|  |  | ANN | 64 | 0.52 | 0.14 | 0.16 | 0.28 |
|  | CD ≥ 36 | GNB | 61 | 0.42 | 0.09 | 0.04 | 0.17 |
|  |  | SVM | 39 | 0.23 | 0.06 | -0.33 | 0.00 |
|  |  | ANN | 57 | 0.32 | 0.07 | -0.10 | 0.09 |
| Fe | SD ≥ 16 | GNB | 84 | 0.71 | 0.37 | 0.45 | 0.54 |
|  |  | SVM | 83 | 0.71 | 0.60 | 0.38 | 0.48 |
|  |  | ANN | 86 | 0.70 | 0.32 | 0.48 | 0.56 |
|  | CD ≥ 36 | GNB | 79 | 0.59 | 0.22 | 0.29 | 0.39 |
|  |  | SVM | 50 | 0.62 | 0.23 | 0.03 | 0.21 |
|  |  | ANN | 79 | 0.55 | 0.25 | 0.29 | 0.39 |
